# Engineering Electro- and Photocatalytic Carbon Materials for CO_2_ Reduction by Formate Dehydrogenase

**DOI:** 10.1021/jacs.2c04529

**Published:** 2022-07-28

**Authors:** Vivek M. Badiani, Carla Casadevall, Melanie Miller, Samuel J. Cobb, Rita R. Manuel, Inês A. C. Pereira, Erwin Reisner

**Affiliations:** † Yusuf Hamied Department of Chemistry, University of Cambridge, Lensfield Road, Cambridge, CB2 1EW, U.K.; ‡ Cambridge Graphene Centre, University of Cambridge, Cambridge, CB3 0FA, U.K.; § Instituto de Tecnologia Química e Biológica António Xavier (ITQB NOVA), Universidade NOVA de Lisboa, Av. da República, 2780-157 Oeiras, Portugal

## Abstract

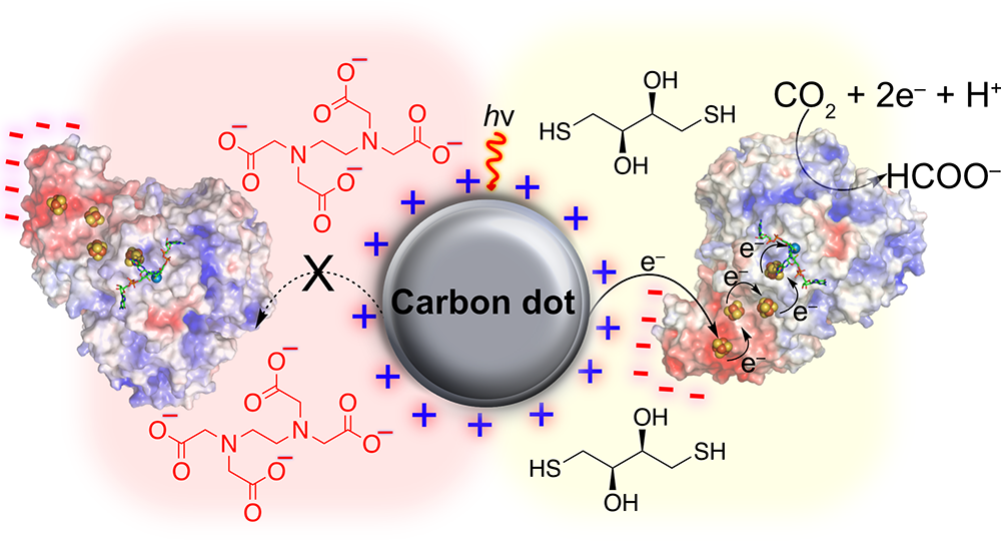

Semiartificial approaches to renewable fuel synthesis exploit the integration of enzymes with synthetic materials for kinetically efficient fuel production. Here, a CO_2_ reductase, formate dehydrogenase (FDH) from *Desulfovibrio vulgaris* Hildenborough, is interfaced with carbon nanotubes (CNTs) and amorphous carbon dots (*a*-CDs). Each carbon substrate, tailored for electro- and photocatalysis, is functionalized with positive (−NHMe_2_^+^) and negative (−COO^–^) chemical surface groups to understand and optimize the electrostatic effect of protein association and orientation on CO_2_ reduction. Immobilization of FDH on positively charged CNT electrodes results in efficient and reversible electrochemical CO_2_ reduction via direct electron transfer with >90% Faradaic efficiency and −250 μA cm^–2^ at −0.6 V vs SHE (pH 6.7 and 25 °C) for formate production. In contrast, negatively charged CNTs only result in marginal currents with immobilized FDH. Quartz crystal microbalance analysis and attenuated total reflection infrared spectroscopy confirm the high binding affinity of active FDH to CNTs. FDH has subsequently been coupled to *a*-CDs, where the benefits of the positive charge (−NHMe_2_^+^-terminated *a*-CDs) were translated to a functional CD-FDH hybrid photocatalyst. High rates of photocatalytic CO_2_ reduction (turnover frequency: 3.5 × 10^3^ h^–1^; AM 1.5G) with dl-dithiothreitol as the sacrificial electron donor were obtained after 6 h, providing benchmark rates for homogeneous photocatalytic CO_2_ reduction with metal-free light absorbers. This work provides a rational basis to understand interfacial surface/enzyme interactions at electrodes and photosensitizers to guide improvements with catalytic biohybrid materials.

## Introduction

The electrocatalytic and solar-driven synthesis of fuels and chemicals from carbon dioxide (CO_2_) provides a sustainable approach to (i) mitigate CO_2_ emissions while (ii) producing energy vectors by storing renewable electricity or solar energy in chemical bonds.^1^ Formate (HCOO^–^) is an attractive product from CO_2_ reduction with a thermodynamic potential similar to that of proton (H^+^) reduction (*E*^0^′_HCOO^–^_ = −0.36 V vs SHE at pH 6.5)^2^ and can be used in fuel cells, chemical synthesis, or as a liquid store for H_2_ via dehydrogenation.^3,4^ Despite much progress in the development of synthetic CO_2_ to formate catalysts, enzymes still serve as benchmarks due to their excellent selectivity, reversibility, and high catalytic rate at moderate overpotentials.^5,6^

**Figure 1:**
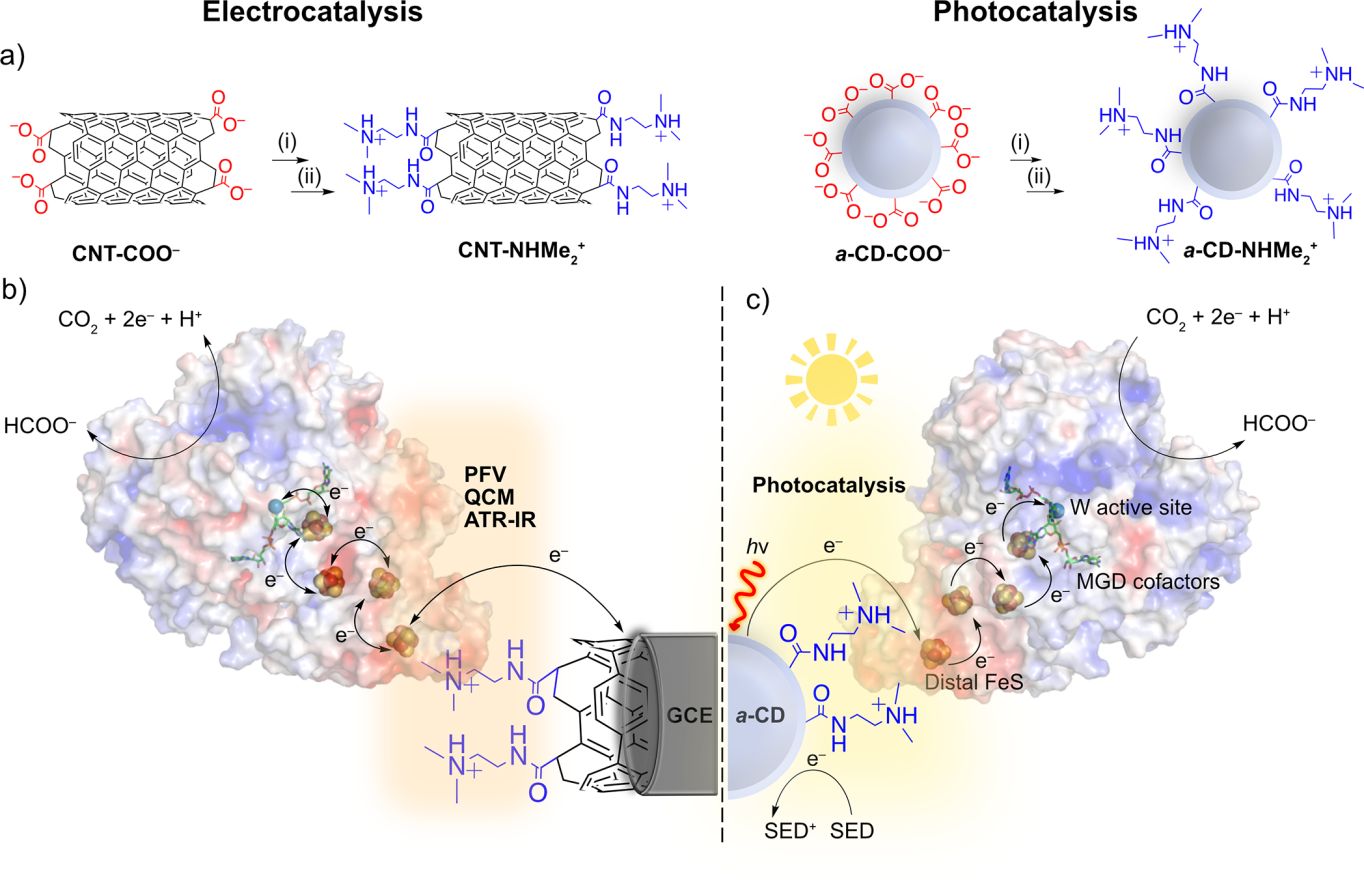
(a) Functionalization of CNT-COO^–^ and *a*-CD-COO^–^ with (i) SOCl_2_, 80 °C, 2 h, and (ii) *N–N*′-dimethylethylenediamine (DMEN), rt, overnight; (b) the use of CNT-NHMe_2_^+^ on a glassy carbon electrode as a scaffold to investigate the oriented immobilization of W-FDH from *Dv*H (pdb: 6SDV) for the reversible electrocatalytic reduction of CO_2_, and (c) the use of *a*-CD-NHMe_2_^+^ as a homogeneous scaffold for the oriented immobilization of FDH for light driven CO_2_ reduction to formate. Electron transfer occurs across the material-enzyme interface via the FeS clusters to the W-active site of FDH, which is coordinated by molybdopterin guanine dinucleotide (MGD) cofactors, resulting in CO_2_ reduction to formate. The oxidized *a*-CD-NHMe_2_^+^ is regenerated by a SED. The electrostatic surface potential of FDH is represented by negatively charged (red) and positively charged (blue) regions.

Formate dehydrogenase (FDH) is the model enzymatic electrocatalyst for the conversion of CO_2_ to formate.^2^ Metal-independent FDHs have been hybridized with photosensitizers for photocatalytic CO_2_ reduction, but viologen-based mediators or stoichiometric amounts of NAD(P)H are required, which are energetically inefficient, toxic, or expensive.^7−13^ On the other hand, metal-dependent FDHs such as molybdenum- and tungsten-containing FDH (Mo/W-FDH) have been established as reversible, mediator-free CO_2_ reduction catalysts on electrodes and have resulted in photoelectrochemical cells for solar fuel synthesis.^14−17^ Specifically, W-FDH from *Desulfovibrio vulgaris* Hildenborough (*Dv*H) presents a W-active site embedded within the protein matrix along with four iron–sulfur (FeS) clusters to facilitate charge exchange between the active site and a suitable redox partner (Figure 1, Figure S1), exhibiting a high CO_2_ reduction turnover frequency (TOF) of 320 s^–1^ in solution assays at pH 6.9.^18,19^

*Dv*H W-FDH was previously interfaced with phosphonated tris(bipyridine)ruthenium(II) (RuP) and diketopyrrolopyrrole (DPP)-sensitized TiO_2_ nanoparticles to demontrate mediator-free photocatalytic CO_2_ reduction to formate using FDH.^20^ Despite the demonstration of photocatalytic CO_2_ reduction using interfacial direct electron transfer (DET), the catalytic rate of the enzyme remained significantly lower than the solution assay activity of W-FDH.^18^ Furthermore, dyes such as RuP and DPP contain either expensive precious metals or are laborious to synthesize, whereas TiO_2_ powder suffers from poor aqueous dispersibility, preventing applications for *in vivo* whole cell photocatalysis.^10,21^ Thus, a homogeneous, fully integrated and mediator-free photocatalytic CO_2_ reduction system with FDH and a scalable light absorber would be desirable.

Photoluminescent carbon dots (CDs) are a low cost, scalable, and homogeneous carbon nanomaterial with applications in bioimaging,^22^ sensing,^23^ and light-emitting devices.^24^ CDs have also been used in photocatalytic H_2_ production using bioinspired synthetic^25^ and enzymatic^26,27^ cocatalysts. The interfacial engineering of CDs with hydrogenases (H_2_ases) has been essential for activity. Specifically, photocatalytic H_2_ evolution with positively charged, amine-terminated CDs was achieved through an interaction with the negatively charged protein surface surrounding the distal FeS cluster of the H_2_ase.^26^ However, enzymatic CO_2_ reduction with functionalized CDs has not yet been accomplished.

Although engineering the material surface is important to realize improvements in activity via optimized physical adsorption,^28^ auxiliary photocatalytic components such as the sacrificial electron donor (SED) and buffer components may also perturb the electrostatic enzyme–material interface, preventing the biohybrid systems from matching the intrinsic enzyme activity.^29,30^

The immobilization of enzymes on modified electrodes (Figure 1a,b)^31^ provides an electrochemical tool to probe the activity of enzyme films (catalytic current) as a function of applied potential, material surface chemistry, and external chemical components (buffers, SEDs, redox mediators), which can guide the improvement in the performance of a photocatalytic support with an analogous surface. W-FDH from *Dv*H has previously displayed DET activity on positively charged amine-modified graphite^32^ and Au^33^ electrodes, but an in-depth understanding of the enzyme–electrode interface and the extension of this observation to photocatalytic materials have not yet been reported (Figure 1a,c).

Here, we develop and study the FDH–carbon interface by electrochemistry to establish the enzyme as an efficient catalyst for photocatalytic CO_2_ reduction to formate (Figure 1). First, FDH is immobilized on functionalized carbon nanotube (CNT) electrodes, and protein film voltammetry (PFV) and chronoamperometry (CA) are used to investigate the effect of surface chemistry on electron transfer. FDH–CNT films are studied by quartz crystal microbalance (QCM) analysis and attenuated total reflection infrared (ATR-IR) spectroscopy to provide insight into the binding and structural integrity of the protein upon immobilization (Figure 1b). Finally, the translation of the ideal surface charge to amorphous CDs (*a*-CDs) allows the complex effects of SEDs and redox mediators on the electro- and photocatalytic activity of the biohybrid to be understood, bridging electrochemistry and photocatalysis and guiding the system toward benchmark metal-free photocatalytic CO_2_ reduction activities (Figure 1c).

## Results and Discussion

### Synthesis and Characterization of CNTs and *a*-CDs

Details for the synthesis of carboxylic acid (−COOH) and tertiary amine (−NMe_2_) CNTs and *a*-CDs based on a previously reported procedure can be found in the Experimental Section.^26,34,35^

Fourier transform infrared spectroscopy (FT-IR) confirms the conversion of *a*-CD-COOH via the loss of the C=O stretching frequency at 1701 cm^–1^, and the introduction of an amide C=O stretch at 1654 cm^–1^ with an additional N–H bending mode at 1546 cm^–1^ (Figure S2), in agreement with previously reported results.^26^ UV–visible (UV–vis) spectroscopy displays a shift in the absorption onset to longer wavelengths upon functionalization with −NMe_2_ (Figure S3), whereas ^1^H NMR spectroscopy shows the presence of two sets of multiplets (2.5 and 2.9 ppm, ethylene protons) and further multiplets (2.3–2.4 ppm, methyl protons) for *a*-CD-NMe_2_ (Figure S4), consistent with previous reports.^26,36^

High-resolution X-ray photoelectron spectroscopy (XPS) (Figure S5) was carried out to confirm the functionalization of the CNTs. Deconvolution of the N 1s peak of CNT-NMe_2_ confirmed the presence of a N–C=O amide at 398.2 eV (1.1%), −NMe_2_ amine at 399.5 eV (1.1%), and nitride at 397.2 eV (2.9%) (Figure S5b) consistent with previous assignments for carbon materials.^37,38^ The percentages provided in the XPS analysis are for the area of each deconvoluted functional group as a percentage of the total sum of the areas of the C 1s, O 1s, and N 1s peaks from the survey spectra. Elemental analysis confirmed an increase in nitrogen content for both *a*-CD-NMe_2_ and CNT-NMe_2_ (Table S1). For CNT-COOH, only pyridinic nitrogen was observed to a small extent (<1%) (Figure S5b, bottom panel), possibly from the incorporation of N atoms into defect sites from the HNO_3_/H_2_SO_4_ oxidation procedure as previously observed.^39,40^

Zeta (ζ) potential measurements confirm a positively charged surface within the physiological pH range (pH 7) for *a*-CD-NMe_2_ (+17 mV) and CNT-NMe_2_ (+9 mV), respectively, whereas negative ζ values were obtained for *a*-CD-COOH (−17 mV) and CNT-COOH (−23 mV), respectively (Figure S6). As such, the samples will be denoted as CNT/*a*-CD-NHMe_2_^+^ and CNT/*a*-CD-COO^–^ throughout this study to describe their ionic character under the employed experimental conditions. A slightly lower ζ value for CNT-NHMe_2_^+^ is likely due to the lower number of functional groups as observed by elemental analysis (Table S1) and high aspect ratio compared with *a*-CD-NHMe_2_^+^.

This characterization supports a similarly functionalized surface for both *a*-CDs and CNTs, where the CNTs are used as an electrocatalytic interface to probe the effect of surface charge on FDH for DET by PFV and CA.

### Protein Film Electrochemistry of FDH on CNTs

In W-FDH electrons are exchanged with the buried active site via four FeS clusters (Figure 1). The interfacial electron exchange site is the outermost (distal) FeS cluster, the protein surface of which is decorated with negatively charged aspartic (Asp) and glutamic (Glu) acid residues (Figure S1, Table S2).^32,33^ According to Marcus theory, DET is only efficient at short distances (<14 Å) between the electrode and FeS cluster; therefore, control over the orientation of FDH upon immobilization is key.^41^ Surface charge can also affect the reorganization energy of electron transfer, which in density of states dependent Marcus theory affects the maximal rate of electron transfer in addition to redox site distance.^42,43^ Therefore, effects of surface charge on the reorganization energy may also offer improvements in the performance of electro- and photocatalytic enzyme systems.

Dispersions of CNT-COO^–^ and CNT-NHMe_2_^+^ were drop-cast onto a precleaned glassy carbon electrode and dried under vacuum to yield a CNT film (thickness ≈ 3.3 μm as measured by scanning electron microscopy (SEM; Figure S7)). FDH (40 pmol; activated by dl-dithiothreitol (DTT)) was drop-cast onto the CNT film to give the CNT|FDH electrode (see Experimental Section).

**Figure 2:**
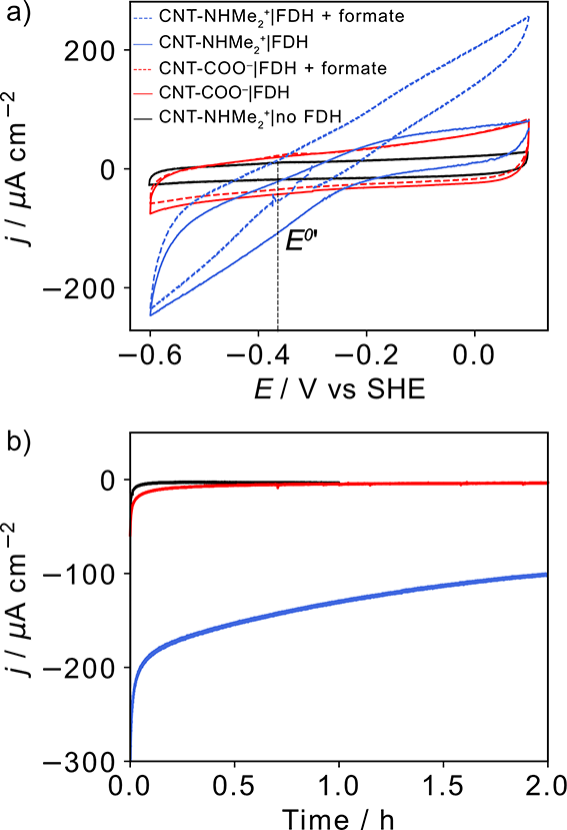
(a) PFV scans of FDH immobilized on CNT-COO^–^ (red traces) and CNT-NHMe_2_^+^ (blue traces) showing CO_2_ reduction only in 1 atm CO_2_ (solid trace) and reversible CO_2_ reduction and formate oxidation in 1 atm CO_2_ and 20 mM sodium formate (dashed trace), (b) CA of the electrodes over 2 h at an *E*_app_ of −0.6 V vs SHE. Conditions: CNT (15 μg) on a glassy carbon electrode (area = 0.071 cm^2^), FDH (1 μL, 40 μM), DTT (1 μL, 50 mM in 50 mM MOPS pH 7), CO_2_-saturated NaHCO_3_/KCl electrolyte solution (100 mM, 50 mM, pH 6.7), sodium formate (20 mM; dashed trace), ν = 5 mV s^–1^, ω = 2000 rpm, 25 °C. The black trace shows the background current of an FDH-free CNT-NHMe_2_^+^ electrode. The vertical black dotted line in (a) denotes the thermodynamic potential (*E*^0^′) for CO_2_/HCOO^–^ estimated from the zero current potential.

PFV scans of CNT-NHMe_2_^+^|FDH in a CO_2_-saturated NaHCO_3_/KCl (100 mM/50 mM, pH 6.7) electrolyte solution demonstrated a respectable current density for CO_2_ reduction (*j*_red_), reaching −247 μA cm^–2^ at −0.6 V vs SHE at pH 6.7 (Figure 2a, solid blue trace). Addition of sodium formate to the CO_2_/NaHCO_3_ containing electrolyte solution resulted in reversible CO_2_/formate interconversion of the CNT-NHMe_2_^+^|FDH electrode, with the formate oxidation current density (*j*_ox_) reaching +246 μA cm^–2^ at +0.1 V vs SHE (Figure 2a, dashed blue trace). Conversely, protein film voltammograms of CNT-COO^–^|FDH (Figure 2a, red trace) and bare CNT-COO^–^ (Figure S8) displayed negligible catalytic current response in the presence of CO_2_ or formate, suggesting the presence of negligible electroactive FDH on the negatively charged CNTs.

The soluble redox mediator methyl viologen (MV^2+^; *E*^0^′ = −0.45 V vs SHE at pH 7)^44^ is used to transfer electrons from the electrode to the distal FeS cluster site regardless of the distance between them. As such, addition of MV^2+^ (0.25 mM) to the electrolyte of CNT-COO^–^|FDH resulted in a mediated electron transfer current (*j*_MET_) of −615 μA cm^–2^ at −0.6 V vs SHE (Figure S9a). This observation suggests that the enzyme is still active but possibly misoriented on the negatively charged CNT film due to electrostatic repulsion of the distal FeS cluster.

The shape of the protein film voltammogram of CNT-NHMe_2_^+^|FDH (Figure 2a, blue trace) displays a linearly increasing current response with increasing potentials, which suggests that there is still a dispersion of FDH electron transfer rates on the positively charged electrode surface.^45^ The addition of MV^2+^ to the electrolyte of CNT-NHMe_2_^+^|FDH confirmed this by yielding an increase over the DET current (*j*_DET_) from −223 μA cm^–2^ to a *j*_MET_ of −1224 μA cm^–2^ at −0.6 V vs SHE (Figure S9b), implying that not all FDH molecules are engaged in DET upon interfacial engineering.

CA at a constant applied potential (*E*_app_) of −0.6 V vs SHE in CO_2_-saturated NaHCO_3_/KCl (100/50 mM, pH 6.7) generated a relatively stable *j*_DET_ for CNT-NHMe_2_^+^|FDH over 2 h, producing 1.25 ± 0.3 μmol cm^–2^ of formate detected by ion chromatography (IC) with a Faradaic efficiency (FE) of >90% (Figure 2b). The decay of 40% of catalytic activity during CA is due to film loss, most likely nondesorptive inactivation processes such as protein unfolding, reorientation, or degradation, as has been previously suggested for FDH, H_2_ase, and bilirubin oxidase on positive and negatively charged self-assembled monolayer (SAM)-Au electrodes.^33,46,47^ A similar current decay of 66% was reported for *Dv*H W–FDH on graphite after 90 min at −0.66 V vs SHE.^32^ No significant Faradaic *j*_DET_ was observed for CNT-COO^–^|FDH or an enzyme-free CNT-NHMe_2_^+^ electrode at *E*_app_ = −0.6 V vs SHE over 2 h as formate was not detectable in the electrolyte solution after CA (Figure 2b).

The high catalytic *j*_DET_ observed for CNT-NHMe_2_^+^|FDH may be assigned to the oriented binding of FDH near the distal FeS cluster, enabling DET. However, as this is governed by electrostatic interactions, exposure of the enzyme-electrode to a charged chemical species could affect the orientation. Good’s buffers such as 3-(*N*-morpholino)propanesulfonic acid (MOPS) are zwitterionic at a pH below the p*K*_a_ of the morpholine nitrogen (p*K*_a_ = 7.2) and is unlikely to screen electrostatic charges between the enzyme and electrode at pH 7. Ethylenediaminetetraacetic acid (EDTA), however, is a commonly used SED in photocatalysis, comprising of four carboxylates (all p*K*_a_ < 3) and two amines (p*K*_a,1_ = 6.16 and p*K*_a,2_ = 10.24), and is therefore negatively charged at pH 7. Drop-casting FDH and immediately adding EDTA (10 mM, pH 7) on the CNT-NHMe_2_^+^ electrode resulted in the decrease of electrocatalytic activity from −248 ± 2 μA cm^–2^ to −117 ± 8 μA cm^–2^ at −0.6 V vs SHE (Figure S10). This reveals that the presence of a charged SED could perturb the enzyme–material interface by competitively binding to CNT-NHMe_2_^+^. The implication of this observation with CDs is discussed in the photocatalysis section below.

### QCM, ATR-IR, and Electron Transfer Studies of FDH on CNTs

QCM and ATR-IR spectroscopy were used to improve our understanding of the nature of the binding and conformation of FDH on positively and negatively charged CNTs. First, a membrane transfer procedure was employed to deposit thin, homogeneous, and reproducible CNT-COO^–^ and CNT-NHMe_2_^+^ films on a gold-coated quartz crystal and a Si ATR-IR prism (Figures S11, S12; thickness ≈ 76 nm as measured by SEM).^48^

**Figure 3:**
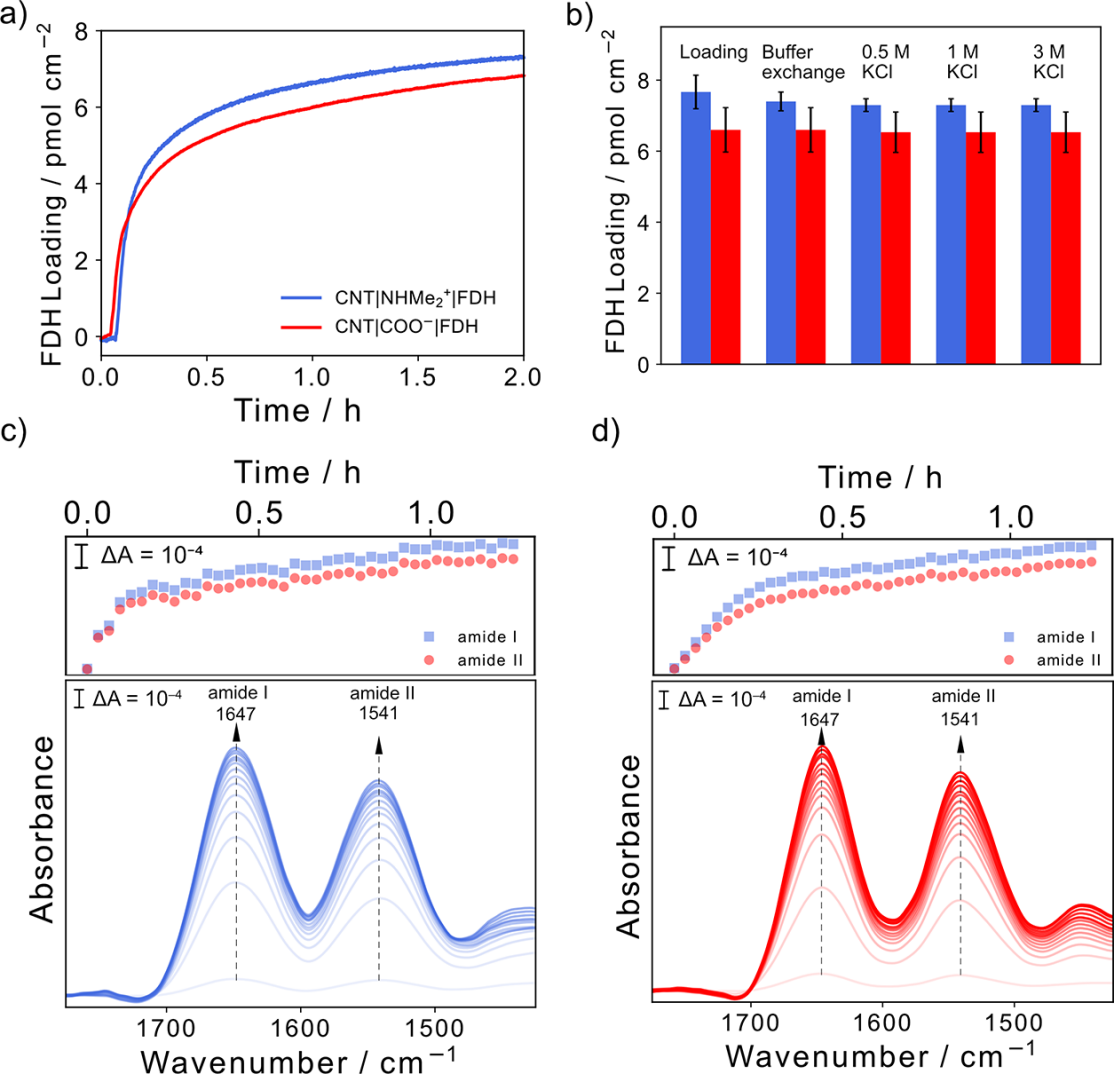
(a) QCM analysis of the adsorption of FDH on CNT-COO^–^ and CNT-NHMe_2_^+^-coated gold-quartz chips. (b) Desorption profiles of FDH on each CNT-coated chip after exposure to increasing concentrations of KCl solution. Conditions: 66 nM FDH, 50 mM MOPS, pH 7, 25 °C, N_2_ atmosphere, 0.141 mL min^–1^ flow rate. ATR-IR absorbance spectra evolution of the amide I and II band region of FDH adsorbed on (c) CNT-NHMe_2_^+^ and (d) CNT-COO^–^ coated on a Si prism waveguide. Each spectrum shown from light to darker shades corresponds to ∼5 min 30 s of time evolved. Conditions: 400 nM FDH, 50 mM MOPS, total volume = 200 μL, pH 7, 25 °C. Error bars represent the standard deviation for a sample size of *n* = 3.

For QCM, after obtaining a stable baseline in enzyme-free MOPS buffer (50 mM, pH 7), FDH (66 nM in 50 mM MOPS, pH 7) was circulated over the CNT-QCM crystals. Loadings of 7.7 ± 0.47 pmol cm^–2^ and 6.6 ± 0.63 pmol cm^–2^ FDH were obtained after 2 h for CNT-NHMe_2_^+^ and CNT-COO^–^, respectively, with the majority of adsorption occurring in the first 20 min, followed by a slower gradual increase in loading (Figure 3a). This may suggest that the porosity of the electrode is inhomogeneous where the enzymes slowly penetrate through the CNT membrane over time.

SEM images of the CNT-coated QCM chip (Figure S11f,g) confirmed the presence of interwoven, flat CNTs, with few pores large enough for FDH to penetrate through the CNT network. After 2 h the amount of bound FDH was on the same order of magnitude as FDH on SAM-modified planar Au^32,33^ and planar TiO_2_,^20^ as quantified by QCM, confirming that for a FDH biomolecule with a diameter of ∼9 nm the CNT membrane is most likely planar/mat-like rather than extensively porous.^18^ Although this morphology does not provide a high surface area, it is well suited for studying the orientation of proteins as it provides limited points of contact, which is also expected for the surface of *a*-CDs in photocatalysis, and provides further evidence that the absence of catalytic current observed for FDH on CNT-COO^–^ could be due to orientation. Furthermore, the Sauerbrey equation used to quantify protein adsorption (Equation S1) is only valid for rigid and evenly distributed layers of biomolecules and, thus, remains acceptable in measurements where close to a monolayer of protein is detected.^49,50^

It was observed above that using a diffusional redox mediator led to a *j*_MET_ of CNT-NHMe_2_^+^|FDH 2-fold higher than CNT-COO^–^|FDH (Figure S9). Furthermore, QCM displayed a similar coverage of FDH on CNT-NHMe_2_^+^ and CNT-COO^–^ (Figure 3a). Therefore, the observed differences in *j*_MET_ may be indicative of a shorter diffusional distance of the mediator or immobilization of the enzyme in an intrinsically more active conformation on CNT-NHMe_2_^+^, further highlighting the importance of surface charge on the design of effective enzymatic CO_2_ reduction systems.

After loading of FDH, the binding strength was quantified by exposure of the QCM crystals to successive ionic concentrations of KCl. No desorption of FDH was observed after rinsing both CNT-NHMe_2_^+^ and CNT-COO^–^ with 3 M KCl (Figure 3b), indicating that the binding is likely to be due to additional noncovalent interactions such as hydrophobic (from the basal plane) or hydrogen bonding (from the −COO^–^ or −NHMe_2_^+^ functional group).^20,33,51^ Furthermore, the integrity of the protein after exposure to 3 M KCl was confirmed by circular dichroism spectroscopy (Figure S13). KCl was also shown to have no effect on solution assay formate oxidation activity but had some effect on CO_2_ reduction (Figure S14).

To further confirm the structural integrity of FDH upon adsorption, a Si ATR-IR prism coated with either the positive or negatively charged CNT film was used (Figure S11d,e). In ATR-IR, reflection of the IR beam results in an evanescent wave penetrating only ∼500 nm normal to the surface of the Si prism, enabling surface-selective detection of the secondary structure of surface-bound enzymes.^21^ As such, the thin nature of the assembled CNT membrane (∼76 nm thickness; Figure S12) allows the immediate detection of FDH upon adsorption on the top surface of the CNT film.

The evolution of amide I and II bands of the protein secondary structure at 1647 and 1541 cm^–1^, respectively, confirms adsorption of FDH to both CNT-COO^–^ and CNT-NHMe_2_^+^ (Figure 3c,d, bottom panel). The adsorption kinetics of the protein are followed by plotting the amide I and II band intensities over time. The trend agrees well with the loading profile observed by QCM, with the majority of loading occuring in the first 20 min followed by a gradual increase in amide band intensity due to the inhomogeneous porosity of the CNT film (Figure 3c,d, top panel). The protein secondary structure is retained regardless of CNT charge as evidenced by the amide I and II band shapes, with no visible broadening or shifts when FDH is adsorbed on either CNT-NHMe_2_^+^ or CNT-COO^–^ (Figure S15), supporting the absence of major conformational changes in the protein structure for both CNT films. For comparison, the ATR-IR spectra of denatured FDH (95 °C, 15 min) showed significant broadening of the amide I band upon loss of the secondary structure (Figure S16).^52^

In the absence of substrate (CO_2_ and HCOO^–^) a nonturnover related peak was observed in the PFV of FDH on CNT-NHMe^2+^ at −0.06 V vs SHE in a 2-(*N*-morpholino)ethanesulfonic acid (MES; 0.1 M, pH 6.5, N_2_) buffer solution (Figure S17a), which was not detectable in the PFV scans in the presence of substrate (Figure 2a). Although the potential is ∼+300 mV more positive than the equilibrium potential of CO_2_/HCOO^–^, a similarly high potential distal FeS cluster was observed for an O_2_-tolerant [NiFe]-H_2_ase from *Aquifex aeolicus*, the redox potential of which may be fine-tuned by the surrounding amino acid environment and intersubunit protein–protein interactions.^53^ However, we cannot unequivocally confirm the identity of this signal without more detailed studies.

Nevertheless, the signal is related to electron transfer with the protein and has therefore been used to determine the electroactive loading and electron transfer properties of the immobilized enzyme. From linear regression of the peak height an electroactive surface coverage of 10.4 ± 0.4 pmol cm^–2^ was estimated (Figure S17a,b), slightly higher than the QCM loading (7.7 ± 0.5 pmol cm^–2^). The different loading density is expected from the increased CNT thickness and related surface area increases of the drop-casted PFV electrode (∼3.3 μm) compared with the CNT membrane on the QCM chip (∼76 nm). From Laviron analysis^54^ an electron transfer rate constant (*k*_ET_) of 9.7 ± 0.5 s^–1^ (Figure S17c) was determined for the enzyme undergoing DET, highlighting possible electron transfer limitations on the electrode when compared to the solution assay activity of FDH.^18^ For comparison, flavin adenine dinucleotide exhibited a *k*_ET_ of 7.6 s^–1^ on CNT electrodes.^55^ It should also be noted that FDH from *Dv*H displays a strong catalytic bias for formate oxidation in solution assays^18^ (Figure S14), whereas an identical CO_2_ reduction and formate oxidation dependence with overpotential is observed by PFV (Figure 2a), which could also be indicative of an electron transfer rate limitation.

The analysis presented here suggests that the charge of the electrode and thus distance of the distal FeS cluster from the electrode surface may be critical for the catalytic activity of FDH, which can be further applied to photocatalytic particle-based systems.

### Photocatalytic CO_2_ Reduction with CD-FDH

**Figure 4:**
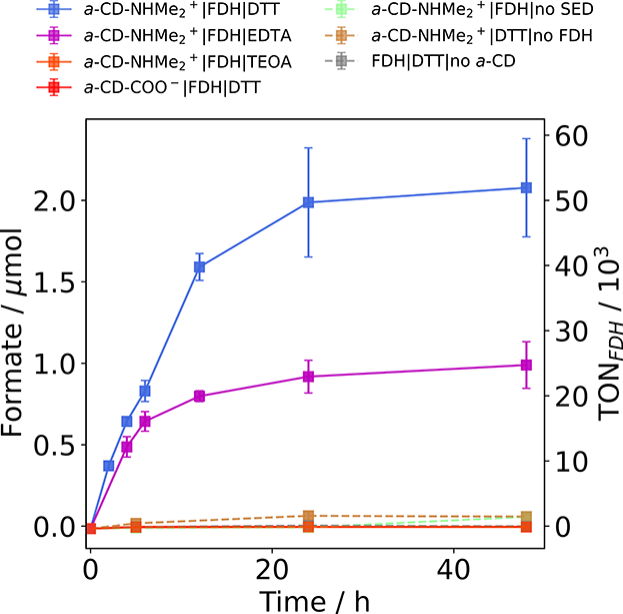
Photocatalytic CO_2_ reduction to formate with FDH immobilized on *a*-CD-COO^–^ (red) or *a*-CD-NHMe_2_^+^ (other colors). Conditions: 40 pmol of FDH, 10 mM SED, 1 mg of *a*-CDs, CO_2_-saturated 100 mM aqueous NaHCO_3_, pH 6.7, 25 °C, total volume = 1 mL, assembled in an anaerobic glovebox, simulated solar-light irradiation: AM 1.5G, 100 mW cm^–2^. In all cases, DTT was used as the SED except for the magenta, orange, and green traces where EDTA, TEOA or no SED was used, respectively. Exclusion control experiments without FDH and *a*-CDs are shown in brown and gray, respectively. Error bars represent the standard deviation for a sample size of *n* = 3.

After establishing the beneficial electron transfer and strong binding of FDH to CNT-NHMe_2_^+^, FDH was interfaced with photoluminescent *a*-CD-NHMe_2_^+^ to investigate its potential for mediator-free, homogeneous photocatalytic CO_2_ reduction to formate (Figure 4). The photocatalytic system was typically assembled by dissolving *a*-CDs (1 mg) in a solution of CO_2_-purged NaHCO_3_ (100 mM, pH 6.7) and the relevant electron donor (10 mM), after which preactivated FDH (1 μL, 40 μM) was added to the borosilicate glass vessel to make up a total volume of 1 mL. The headspace of the vial was then purged with CO_2_, sealed, and irradiated (AM 1.5G, 100 mW cm^–2^), and the amount of formate produced was monitored by IC. Approximately 25 μM of DTT is present in all photocatalytic reactions from the activation of FDH.

EDTA, triethanolamine (TEOA), and DTT were employed as SEDs (10 mM in 100 mM NaHCO_3_, pH 6.7) and assessed for their photocatalytic activity with *a*-CD-NHMe_2_^+^|FDH.^56,57^ No formate was detected in the presence of TEOA, whereas the system using EDTA generated 0.92 ± 0.1 μmol of formate, giving a turnover number (TON) of (23 ± 3) × 10^3^ mol of formate (mol FDH)^−1^ and a TOF of (0.96 ± 0.13) × 10^3^ h^–1^ during 24 h of irradiation. In the *a*-CD-NHMe_2_^+^|FDH system, DTT is used as a chemical reducing agent (*E*^0^′_DTT_ = −0.33 V vs SHE at pH 7)^58^ to activate the enzyme.^18^ When DTT was employed at higher concentrations (10 mM) to serve as a SED, a more than 2-fold increase in photocatalytic activity was observed compared to EDTA, producing 1.98 ± 0.34 μmol of formate with a TON of (49.5 ± 8.5) × 10^3^ mol formate (mol FDH)^−1^ after 24 h and a TOF of (2.1 ± 0.3) × 10^3^ h^–1^ after 24 h of irradiation, which is comparable to the previously reported *a*-CD-NHMe_2_^+^|H_2_ase system for H_2_ production (1.8 × 10^3^ h^–1^ after 24 h).^26^

To rationalize these observations, we first considered the redox potentials of the SEDs. EDTA and TEOA have similar redox potentials of ∼+0.8 V vs SHE (at pH 7)^59,60^ whereas DTT has a potential of −0.33 V vs SHE (at pH 7).^58^ Although DTT has a much higher reducing power, all of the employed SEDs are far more negative than the valence band of *a*-CDs (conduction band ∼–0.8 V vs SHE, valence band ∼+1.7 V vs SHE)^61^ and thus all possess sufficient thermodynamic driving force to proceed via similar electron transfer processes to quench the excited hole state of the *a*-CDs.

Instead, we turn our attention to the electrostatic charge of the SEDs under the photocatalytic conditions (pH 6.7). TEOA (p*K*_a_ = 7.74) is likely to be protonated at pH 6.7, forming TEOAH^+^.^62^ Attractive interactions between TEOAH^+^ and *a*-CD-NHMe_2_^+^ are therefore impeded by a positive-positive electrostatic repulsion, which could limit efficient electron donation. The role of SED-photosensitizer interactions for efficient hole quenching was previously reported between TEOA and melamine-functionalized carbon nitride for photocatalytic H_2_ evolution.^63^ Additionally, TEOAH^+^ may shield the negatively charged Glu/Asp sites on FDH near the distal FeS cluster, inhibiting efficient interfacing with *a*-CD-NHMe_2_^+^ and preventing photocatalytic activity.^27^ On the other hand, EDTA is predominantly deprotonated at pH 6.7. In the presence of *a*-CD-NHMe_2_^+^, the negatively charged EDTA species will engage in negative–positive attractive electrostatic interactions with the photosensitizer, facilitating efficient electron transfer and thus enabling photocatalytic conversion of CO_2_. However, these commensurate interactions between *a*-CD-NHMe_2_^+^ and EDTA may prevent the binding of FDH in an electroactive orientation by shielding the surface charge of the *a*-CDs, which may be the reason for the reduced *j*_DET_ observed upon coaddition of EDTA with FDH on the CNT-NHMe_2_^+^ electrode (Figure S10).

EDTA has previously been shown to influence the surface charge of cadmium sulfide nanoparticles^64^ and zeta potential measurements confirm a decrease in the ζ value from +22 ± 4 mV to +3.8 ± 0.8 mV when EDTA was added to *a*-CD-NHMe_2_^+^ in NaHCO_3_/CO_2_ (100 mM, pH 6.7), whereas the ζ value of *a*-CD-NHMe_2_^+^ in the presence of TEOA was +13.2 ± 1.9 mV (Figure S18), confirming that the interaction of EDTA with *a*-CD-NHMe_2_^+^ shields the surface charge. The p*K*_a_ of DTT is 9.62,^65^ and it will thus remain neutral and unlikely to interact with the *a*-CD-NHMe_2_^+^ surface and FDH via strong electrostatic interactions. The unperturbed *a*-CD-NHMe_2_^+^ surface in the presence of DTT was confirmed with a measured ζ value of +17.2 ± 0.6 mV (Figure S18). By maintaining the positive charge of *a*-CD-NHMe_2_^+^, a higher fraction of FDH molecules might orient via the distal FeS cluster, leading to the high activities for FDH photocatalysis reported in this work (Table S3).^7,11,12,20,66−68^

The strong binding of the enzyme to CNT-NHMe_2_^+^ observed by QCM (Figure 3b) revealed that exposure of the preformed biohybrid to ionic species (K^+^ and Cl^–^) is unlikely to desorb immobilized FDH due to the possible presence of other noncovalent interactions such as hydrogen bonding.^33^ Therefore, the order of assembly of the biocatalytic systems in the presence of a charged electron donor (EDTA^–^ and Na^+^) is likely to be critical in both electro- and photocatalysis. In separate photocatalytic experiments, a 61% increase in formate production activity was observed when FDH was incubated with *a*-CD-NHMe_2_^+^ before the addition of EDTA to allow initial binding of the enzyme and photosensitizer prior to any perturbation of the *a*-CD surface by the negatively charged EDTA (Figure S19). These results confirm the strong nature of the FDH interaction with −NHMe_2_^+^ both on CNT electrodes and in solution with *a*-CD-NHMe_2_^+^, supporting the previously observed QCM experiments (Figure 3b).

Optimal photocatalytic CO_2_ reduction was observed with 1 mg mL^–1^*a*-CD-NHMe_2_^+^ and 40 nM FDH (Figure S20). The *a*-CD-NHMe_2_^+^ diameter of ∼6.8 nm^26^ is slightly smaller than FDH (diameter ∼9 nm), which results in an expected ratio of CD to FDH of approximately 1:1 under these conditions. Reduced photocatalytic activity was observed at higher *a*-CD concentrations (>1 mg mL^–1^), most likely due to the blocking of light absorption and inefficient charge transfer to FDH (Figure S20),^25^ whereas a lower concentration of *a*-CD (0.5 mg mL^–1^) also led to lower photocatalytic activity, possibly due to less efficient light harvesting (Figure S20).

Exclusion control experiments under optimized conditions included the removal under separate experiments of *a*-CD-NHMe_2_^+^, FDH and the electron donor DTT, which yielded no formate under irradiation (Figure 4), with the latter result confirming that the amount of residual DTT (25 μM) from FDH activation is insufficient to act as an SED. Furthermore, photocatalytic experiments of FDH with *a*-CD-COO^–^ and DTT as the SED generated no detectable formate by IC, corroborating the electrochemical observations of CNT-COO^–^|FDH (Figure 2).

^13^C-Isotopic-labeling studies confirmed that formate was produced from NaH^13^CO_3_/^13^CO_2_ (pH 6.7) with a doublet at δ = 8.35 ppm (*J* = 195 Hz) detected by ^1^H NMR spectroscopy due to the coupling of the ^1^H with the ^13^C (Figure S21).^20,69^ The external quantum efficiency (EQE; Equation S3) was measured by irradiating the optimized sample (*a*-CD-NHMe_2_^+^|FDH in CO_2_-saturated NaHCO_3_/DTT, 100 mM/10 mM, pH 6.7) with monochromatic light at a wavelength of 365 nm and an intensity of 4.9 mW cm^–2^. An EQE of 0.2 ± 0.1% was obtained after 48 h of irradiation, which compares well to [NiFeSe]-H_2_ase interfaced with *a*-CD-NHMe_2_^+^ (0.30%, λ = 365 nm)^26^ and CN_*x*_ (0.07%, λ = 360 nm).^70^

### Quantifying DET in CD-FDH Photocatalysis

To assess the efficiency of FDH photocatalytic DET on CDs, MV^2+^ was used as a soluble redox mediator to transfer electrons to the distal FeS cluster site regardless of distance from the *a*-CDs.^44^

**Figure 5:**
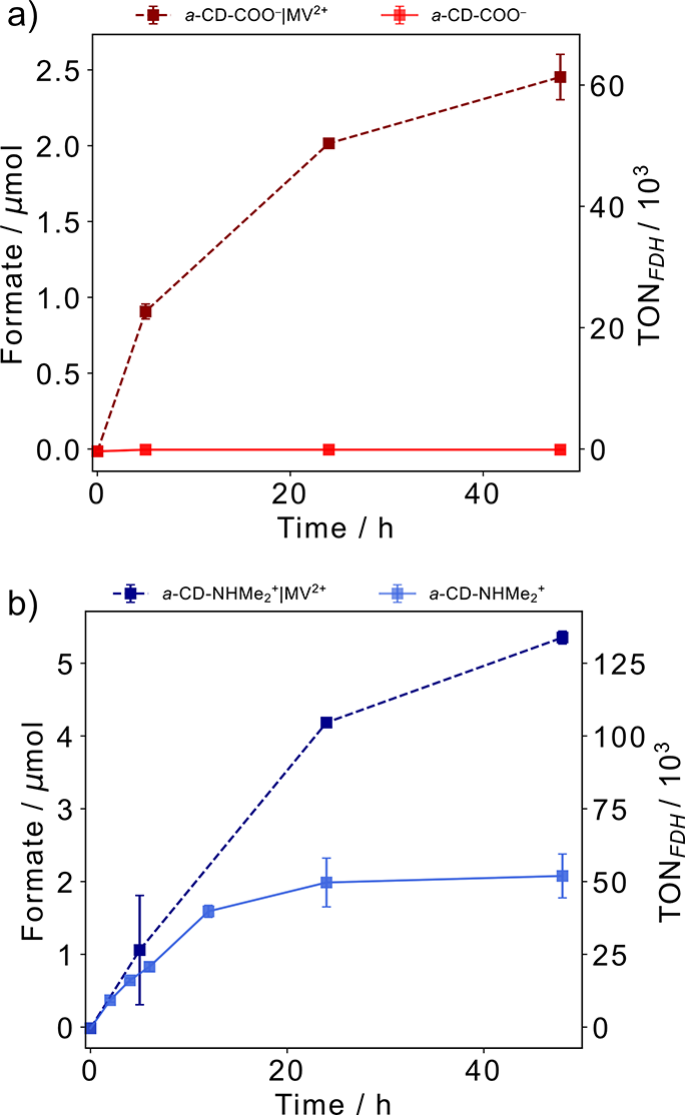
Photocatalytic CO_2_ reduction to formate with FDH assembled with (a) *a*-CD-COO^–^ (red trace) or (b) *a*-CD-NHMe_2_^+^ (blue trace) in the absence (solid trace) or presence of MV^2+^ (dashed trace) during 48 h. Conditions: 40 pmol of FDH, 10 mM DTT, 1 mg mL^–1^*a*-CDs, CO_2_-saturated 100 mM aqueous NaHCO_3_, 1 mM MV^2+^, pH 6.7, 25 °C, total volume = 1 mL, assembled in an anaerobic glovebox, simulated solar-light irradiation: AM 1.5G, 100 mW cm^–2^. Error bars represent the standard deviation for a sample size of *n* = 3.

The addition of MV^2+^ (1 mM) to *a*-CD-COO^–^|FDH led to a substantial increase in mediated photocatalytic formate (formate_MET_) production to 2.01 ± 0.02 μmol after 24 h (Figure 5a) and together with the PFV, QCM, and ATR-IR studies suggests that the enzyme is bound and active but possibly misoriented on the −COO^–^ functional group. Addition of MV^2+^ to *a*-CD-NHMe_2_^+^|FDH also led to an increase in formate production, from 1.98 ± 0.34 μmol to 4.18 ± 0.06 μmol after 24 h (Figure 5b). Like CNT-NHMe_2_^+^|FDH (Figure S9b), this result indicates that not all FDH molecules are engaged in DET on *a*-CD-NHMe_2_^+^. The formate_DET_/formate_MET_ ratio of <1 for *a*-CD-NHMe_2_^+^|FDH is unlikely due to an excess of unbound FDH in solution as optimization experiments did not show an increase in activity at higher *a*-CD-NHMe_2_^+^ concentrations (Figure S20). Similar photocatalytic formate_DET_/formate_MET_ ratios were observed on RuP-TiO_2_ with FDH (0.3),^20^*a*-CD-NHMe_2_^+^|H_2_ase (0.18),^26^ and [FeFe]-H_2_ase on aspartic acid CDs (0.3).^27^

The initial amounts of formate_MET_ for *a*-CD-COO^–^|FDH (0.91 ± 0.05 μmol) and *a*-CD-NHMe_2_^+^|FDH (1.05 ± 0.75 μmol) were similar within 5 h of irradiation. However, *a*-CD-NHMe_2_^+^|FDH produced more formate_MET_ under prolonged irradiation, reaching 5.35 ± 0.08 μmol and a TON of (134 ± 2) × 10^3^ mol of formate (mol FDH)^−1^ after 48 h, which suggests an enhanced stability of FDH when directly wired to the *a*-CDs via the distal FeS.

The results herein demonstrate that despite interfacial engineering, controlling the electron transfer rates of the entire protein population on both electrodes and photosensitizers remains a challenge. For the carbon materials, this is possibly due to the presence of additional functional groups on the CNT-COOH and *a*-CD-COOH starting material (−OH, C=O, epoxides) which could offer uncontrolled immobilization sites.^71^ Alternative methods of enzyme immobilization such as site-specific covalent immobilization^72^ or the use of redox polymers^73^ provide further avenues to attempt to improve the efficiency of the enzyme–material interface.

## Conclusion

We report a redox mediator-free, homogeneous photocatalytic CO_2_ reduction system using FDH. The electrostatic interaction of the negatively charged protein surface in proximity of the distal FeS cluster region of FDH with a −NHMe_2_^+^ functional group on the CNT and CD surface appears central to enabling efficient DET for electro- and photocatalytic CO_2_ reduction to formate. QCM and ATR-IR spectroscopy confirm the binding and structural integrity of FDH on the positively and negatively charged CNT films, and together with the redox mediator MV^2+^, they demonstrate the importance of surface charge for effective DET. Upon direct interfacing of FDH with the electrode, nonturnover electrochemical signals are resolved to provide the electroactive loading of protein and the electron transfer constants. The supramolecular assembly of both CNT-NHMe_2_^+^ and *a*-CD-NHMe_2_^+^ with FDH is shown to be significantly influenced by the presence of charged SEDs, and rational selection of a neutral SED enhances photocatalytic activity 2-fold, resulting in an enzyme hybrid system with benchmark performance. Analysis of MET suggests that higher DET rates are still achievable by further improving the FDH–material interface on carbon allotropes in future development.

## Supporting Information Available

Experimental section (Figures S1–S21, Tables S1–S3); PyMOL analysis of charged amino acids on FDH; FT-IR spectra of a-CDs; UV–vis spectra of *a*-CDs; ^1^H NMR spectra of a-CDs; XPS survey spectra, N 1s, O 1s, and C 1s spectra of CNTs; zeta potential of CNTs and *a*-CDs; SEM images of CNT films for PFV; optical and SEM images of the CNT membrane transfer procedure; circular dichroism of FDH after 3 M KCl exposure; solution assay activities of FDH after 3 M KCl exposure; ATR-IR analysis of amide bands; ATR-IR analysis of denatured FDH; nonturnover analysis of FDH on CNT-NHMe_2_^+^; zeta potential analysis of *a*-CD-NHMe_2_^+^; EDTA pre- and postaddition photocatalysis; EDTA PFVs; *a*-CD-NHMe_2_^+^ concentration optimization; ^13^C labeling; PFV measurements of CNT-FDH electrodes with MV^2+^; table with elemental analysis composition of CNTs and *a*-CDs; table of negatively charged amino acids <14 Å from the distal FeS cluster; table of comparison with state-of-the-art FDH photocatalytic systems (PDF)

## Notes

Raw data related to this article are available at the University of Cambridge data repository: https://doi.org/10.17863/CAM.86570.
