# Supplementary material for: Engineering Electro- and Photocatalytic Carbon Materials for CO2 Reduction by Formate Dehydrogenase
Source: J Am Chem Soc. 2022 Jul 28;144(31):14207–16. doi: 10.1021/jacs.2c04529 (PMC9376922; doi:10.1021/jacs.2c04529)
Supplement: Supplementary file 1 — ja2c04529_si_001.pdf [file ja2c04529_si_001.pdf]

Supplementary material for:

Engineering electro- and photocatalytic carbon materials for CO_2 reduction by formate dehydrogenase

Vivek M. Badiani,[†,‡] Carla Casadevall,[†] Melanie Miller,[†] Samuel J. Cobb,[†] Rita R. Manuel,[§] Inês A. C. Pereira,[§] and Erwin Reisner[*,†]

† Yusuf Hamied Department of Chemistry, University of Cambridge, Lensfield Road, Cambridge, CB2 1EW, U.K.

‡ Cambridge Graphene Centre, University of Cambridge, Cambridge, CB3 0FA, U.K.

§ Instituto de Tecnologia Química e Biológica António Xavier (ITQB NOVA), Universidade NOVA de Lisboa, Av. da República, 2780-157 Oeiras, Portugal.

Experimental section

Chemicals and Materials. The following chemicals and materials were obtained from commercial suppliers and used without further purification unless otherwise stated: multiwalled carbon nanotubes, carboxylic acid functionalized (CNT-COOH, Sigma Aldrich, >8% functionalized, L 9.5 nm × 1.5 µm), citric acid (Sigma Aldrich, ≥99.5%), thionyl chloride ($SOCl_2$, Sigma Aldrich, 97%), tetrahydrofuran (anhydrous, THF, Sigma Aldrich, 99.9%), *N*-methyl-2-pyrrolidone (NMP, anhydrous, Sigma Aldrich, 99.5%), *N,N'*-dimethylethylenediamine (DMEN, Sigma Aldrich, ≥98 %, dried over 4 Å molecular sieves), alumina powder (Al_2O_3, 0.5 µm, Sigma Aldrich, 99.99 %), ethanol absolute (VWR, ≥99.8%), hydrogen peroxide (H_2O_2, Sigma Aldrich, 33%), sulfuric acid (H_2SO_4, Sigma Aldrich, 99%), methyl viologen dichloride hydrate (MV^{2+}, Sigma Aldrich, 98%), Nafion® (Sigma Aldrich), Parafilm® M (Sigma Aldrich), sodium hydrogen carbonate ($NaHCO_3$, Sigma Aldrich, ≥99.9 %), sodium hydrogen carbonate-^{13}C ($NaH^{13}CO_3$, Sigma Aldrich, 98 atom % ^{13}C), potassium chloride (KCl, Fisher Chemical), rubber septa (Subaseal), Whatman® nitrocellulose membrane filter (Sigma Aldrich, NC 45, pore size 0.45 µm), 3-(*N*-morpholino)propanesulfonic acid (MOPS, Sigma Aldrich, ≥99.5%), sodium hydroxide (NaOH, Sigma Aldrich, ≥97%), hydrochloric acid (HCl, Sigma Aldrich, 37%), DL-Dithiothreitol (DTT, Fisher, ≥98.0%), sodium formate (Sigma Aldrich, ≥99.0%), formate standard for IC (*Trace***CERT**®, 1000 mg/L in water, Sigma Aldrich). Buffer solutions were prepared using water from a Simplicity UV MilliQ system (18.2 MΩ cm at 25°C) and consisted of $NaHCO_3$ (100 mM), KCl (50 mM) and sodium formate (20 mM). Gases (CO_2 and N_2 with 2% CH_4) were supplied by BOC. W-FDH from *Desulfovibrio vulgaris* Hildenborough was expressed, purified and characterized according to a published method.[1] All purification steps were performed under aerobic conditions at 4 °C. 40 µM FDH stock solutions with an activity of 1100 s^{-1} for formate oxidation and 320 s^{-1} for CO_2 reduction were stored in a buffer solution (20 mM Tris-HCl, 10% glycerol, 10 mM $NaNO_3$, pH 7.6) at −40 °C under N_2 atmosphere. All measurements with FDH were carried out in an anaerobic glovebox (MBraun, N_2 atmosphere, < 0.1 ppm O_2). Potentials for the electrostatic surface contours of enzymes were calculated with the APBS Electrostatics plugin [https://server.poissonboltzmann.org/pdb2pqr] with correction for charges of the FeS clusters, Selenocysteine, Tungsten and MGD cofactors in the active site.[1] PyMOL (version 2.3.4, Schrodinger, LLC) was used for enzyme visualization.

Synthesis of *a*-CD-COO⁻. *a*-CD-COO⁻ was synthesized and characterized as previously described.[2-4] Briefly, citric acid (100 g) was thermolyzed under air at 180 °C for 40 h producing carboxylic acid-capped amorphous CDs as an orange-brown high-viscosity liquid. The CDs were dissolved in water, filtered through a 0.2 μm syringe filter and freeze-dried to yield *a*-CD-COO⁻ as a yellow-orange powder. Microanalysis found: C, 52.64%; H, 5.31%; N, 0.00%.

Synthesis of *a*-CD-NHMe$_2^+$. *a*-CD-NHMe$_2^+$ was synthesized as previously described.[2] First, activation of *a*-CD-COO⁻ (2.0 g) was carried out by refluxing with $SOCl_2$ (60 mL) under continuous stirring at 80 °C under N_2 for 3 h. $SOCl_2$ was removed under reduced pressure and the resulting *a*-CD-COCl were dissolved in dry THF and filtered. The solvent was evaporated under high vacuum to yield acyl chloride capped CDs (*a*-CD-COCl, 1.6 g). *a*-CD-COCl (1.6 g) was stirred with neat DMEN (30 mL, dried over 14 Å molecular sieves) at room temperature in the dark for 3 h, whereupon DMEN was removed under high vacuum. The *a*-CD-NHMe$_2^+$ were dissolved in acetone and filtered through a 0.2 μm syringe filter. Acetone was removed under high vacuum and the product redispersed in methanol and washed (3 × 5 mL) before drying for 12 h under high vacuum at 40 °C. The resulting *a*-CD-NHMe$_2^+$ were then dissolved in water and freeze-dried to yield a light brown hygroscopic oil. Microanalysis found: C, 53.68%; H, 8.73%; N, 14.94%.

Synthesis of CNT-NHMe$_2^+$. Carboxylic acid-capped multiwalled carbon nanotubes (CNT-COO⁻) were purchased from Sigma Aldrich. CNT-COO⁻ (20 mg) was refluxed with $SOCl_2$ (2 mL) at 80 °C under N_2 for 2 h. $SOCl_2$ was removed under reduced pressure and the resulting CNT-COCl were dissolved in dry THF (5 mL) and cannulated. CNT-COCl (20 mg) was stirred with neat dry DMEN (2 mL) at room temperature in the dark overnight, whereupon DMEN was removed under high vacuum. The CNT-NHMe$_2^+$ was dissolved in acetone and cannulated, after which the CNT-NHMe$_2^+$ were redispersed in methanol (3 x 5 mL) and cannulated before drying for 12 h under high vacuum at 40 °C. Microanalysis found: C, 71.06%; H, 4.38%; N, 1.77%.

Preparation of CNT electrodes. CNT-NHMe$_2^+$ (1 mg mL $^{-1}$) and CNT-COO⁻ (1 mg mL $^{-1}$) were dispersed in NMP:H_2O (1:1 *v/v*) and H_2O, respectively by sonicating for 4 h. A glassy carbon electrode (GCE, 0.071 cm^2) was cleaned by abrading with an Al_2O_3 slurry (0.5 μm) for 30 s before sonicating in H_2O and ethanol sequentially for 5 mins each and blow dried with N_2.

Then, 15 µL of the CNT dispersion (15 µg) was drop-cast on the GCE and dried under vacuum for 20 mins to yield a CNT film.

Preparation of CNT|FDH electrodes. FDH (1 µL, 40 pmol) was mixed with DTT (1 µL, 50 mM) in MOPS (50 mM, pH 7) and incubated for 20 min. The FDH-DTT mixture was then diluted with 3 µL of MOPS (50 mM, pH 7) and drop-cast on the CNT-GCE electrode and left to incubate for 1 min after which the electrode was immersed in the electrolyte.

Protein film voltammetry. All protein film voltammetry experiments were carried out in an anaerobic glovebox (MBraun, N_2 atmosphere, <0.1 ppm O_2). A gas-tight two compartment cell with a Nafion® membrane separating the compartments was equipped with a three-electrode setup, consisting of a Ag|AgCl (sat. KCl) reference electrode and quoted with respect to the standard hydrogen electrode (SHE) using the conversion $E_{SHE} = E_{Ag|AgCl} + 0.197$ V (25 °C), alongside a Pt mesh counter electrode and a CNT-$NHMe_2^+$|FDH or CNT-COO^-|FDH rotating disk working electrode (RDE). An electrolyte solution containing $CO_2/NaHCO_3$ (100 mM) and KCl (50 mM) at pH 6.7 was prepared by dissolving $NaHCO_3$ and KCl in H_2O and purging with CO_2 for 15 min. The electrochemical cell was filled with electrolyte (3 mL counter compartment, 6 mL working electrode compartment), sealed with rubber septa, constantly kept at 25 °C with a chiller, and purged again with CO_2 for 5 min before the start of the measurement. All electrochemical experiments were performed with an Ivium CompactStat potentiostat and a Pine Instruments rotating disk electrode rotator. Voltammograms were recorded with a scan rate of 5 mV s^{-1} at a rotation speed (ω) of 2000 rpm. Chronoamperometry was performed at −0.6 V vs SHE. All data processing was performed using Python 3.9.5.

Preparation of CNT-coated QCM and ATR-IR substrates by the membrane transfer method. The CNT membranes were prepared using a modified reported procedure.[5] CNTs (1 mg) were ultrasonicated in 10 mL H_2O for 90 s and added to 240 mL H_2O and bath sonicated for a further 30 min. The resulting dispersion was left overnight to allow large CNT agglomerates to separate from the supernatant. The supernatant (10 mL) was then dispersed in H_2O (100 mL), sonicated for 5 min and the dispersion filtered through a Whatman® nitrocellulose membrane filter (NC 45, pore size 0.45 µm) and left for 30 min under vacuum. Then, the required diameter (QCM = 0.8 cm^2, ATR-IR = 1 cm^2) was cut out and the CNT-filter paper placed face down on the relevant substrate, slightly wet with H_2O and pressed down. The substrate and filter paper were then placed in the oven at 80 °C with a heavy weight

on top of the filter paper and dried for 30 min. The substrate was removed from the oven and cooled to room temperature before being gently dissolved in two acetone baths for 30 min each, and finally a methanol bath for 30 mins to yield the CNT-membrane-covered substrate.

Quartz crystal microbalance. QCM experiments were conducted with a Biolin Q-Sense Explorer module and a custom-designed QCM electrochemical cell in an anaerobic glovebox (MBraun, N_2 atmosphere, < 0.1 ppm O_2). The pre-prepared CNT-QCM chip was loaded into a custom QCM cell (Biolin Scientific), and MOPS (50 mM, pH 7) was circulated over the chip for 30 min until a stable baseline was achieved. FDH (66 nM in 50 mM MOPS, pH 7) was circulated over the QCM chip, and the frequency response observed. Enzyme adsorption was quantified by monitoring changes in the resonance frequency of the piezoelectric quartz chip. The frequency was related to the mass through the Equation S1[6]:

$$\Delta f = -\frac{2{f_o}^2}{A\sqrt{p_q \mu_q}}\Delta m \quad \text{(S1)}$$

where f_0 is the resonance frequency of the quartz oscillator, A is the piezoelectrically active crystal area, Δm is the change in mass, p_q is the density of quartz, and μ_q is the shear modulus of quartz. To convert the mass adsorbed to quantity of enzyme, an assumption was made that 25% of the adsorbed mass consisted of water molecules bound to the enzyme, which was 172.9 kDa for hydrated FDH in weight.[1]

Attenuated total reflection infrared spectroscopy. Attenuated total reflection infrared (ATR-IR) spectroscopy measurements were performed in a single-reflection PIKE ATR-IR setup and a customized ATR-cell using a Si prism with an angle of incidence of 60°. In order to probe FDH immobilization and surface interaction, CNT films were transferred onto the prism as previously described by the membrane transfer method.[5] ATR-IR spectra were recorded from 4000 to 1000 cm^{-1} with a spectral resolution of 4 cm^{-1} on a Bruker Vertex 70 spectrometer equipped with a liquid N_2 cooled photovoltaic MCT detector. 256 scans were co-added for one spectrum, requiring an accumulation time of 1 min 55 s. ATR-IR spectra were evaluated using a custom-made Python script. Immobilization of FDH was accomplished by adding FDH (80 pmol) to a 200 μL MOPS buffer solution (50 mM) at pH 7 for 2 h at room temperature. For measurement of the denatured sample, FDH (80 pmol) was heated at 95 °C for 15 mins with a VWR Dry Mini Block Heater and subsequently measured by ATR-IR.

FDH incubation with KCl. FDH (84 μM) in 20 mM Tris-HCl buffer, pH 7.6, with 10 % (v/v) glycerol, was activated by incubation with DTT (50 mM) for 5 mins. The sample was washed with DTT-free buffer and incubated with KCl (3 M) for 30 mins, after which KCl was removed by washing with the Tris-HCl buffer.

Circular dichroism spectroscopy. Spectra of FDH samples before and after KCl incubation were recorded with 0.102 mg mL^{-1} of protein using a Jasco J-815 spectropolarimeter (Easton, MD, USA) equipped with a Jasco CDF-426S Peltier temperature controller, in a 0.1 cm path quartz cuvette. Spectra were acquired from 200 nm to 260 nm at 20°C, considering three scan accumulations, with a data integration time of 2 s, 50 nm min^{-1} scan rate, and N_2 flow of 8 L min^{-1}. The results include buffer subtraction.

Solution activity assays. FDH was incubated with DTT (50 mM) prior to assaying as previously reported.[1] For formate oxidation, the reduction of benzyl viologen (BV^{2+}; 0.1 mM) was monitored at 555 nm ($\varepsilon_{555\ nm}$ (BV^{+}) = 12 mM^{-1} cm^{-1}) in a potassium phosphate buffer (50 mM, pH 7.6) containing 20 mM sodium formate. For CO_2 reduction, Zn-reduced MV^{2+} (0.1 mM) was oxidized and followed spectroscopically at 578 nm ($\varepsilon_{578\ nm}$ (MV^{+}) = 9.7 mM^{-1} cm^{-1}) in a potassium phosphate/$NaHCO_3$ buffer (50 mM/50 mM, pH 6.9). One unit of reducing activity is defined as the amount of FDH capable of reducing 1 μmol of CO_2 (oxidizing 2 μmol MV^{+}) per min. A final concentration of 1.4 nM enzyme was used. The turnover numbers were calculated considering one catalytic active protomer αβ (138 kDa).

Photocatalysis. FDH (1 μL, 40 μM) was incubated with DTT (1 μL, 50 mM in 50 mM MOPS, pH 7) for 20 min. *a*-CD-$NHMe_2^{+}$ or *a*-CD-COO^{-} (1 mg) was dissolved in $NaHCO_3$ (100 mM, CO_2 purged) with the relevant SED (10 mM) at a total volume of 1 mL, after which the activated FDH was added. The photoreactor vial was sealed and the headspace purged with CO_2 for at least 10 min to give a final pH value of 6.7. The vials were irradiated with a solar light simulator (AM 1.5G) and stirred at 600 rpm. Aliquots (100 μL) were removed at time intervals and diluted in 900 μL H_2O before injection through a 0.2 μm syringe filter into an ion chromatograph (IC) to quantify the amount of produced formate.

Treatment of data. All analytical measurements were performed in triplicate and the data treated by: for a sample of n observations (x_i), the unweighted mean value (x_0) and the standard deviation (σ) with an assumption of a minimum of 10 % were calculated by the equations:

$$x_0 = \sum_i \frac{x_i}{n} \qquad \sigma = \sqrt{\frac{\sum_i (x_i - x_0)^2}{n-1}} \qquad \text{(S2)}$$

External quantum efficiency measurement. Formate production was carried out in an airtight quartz cuvette with stirring (3.89 mL total volume, 1 mL solution volume). The vial was irradiated for 48 h over a fixed area (1 cm^2) with a Xe lamp (LOT) equipped with a monochromator (LOT MSH300) set to λ = 365 nm. The light intensity was measured using an International Light Technologies photometer (ILT1400); I = 4.9 mW cm^{-2}. The EQE was calculated using the following formula:

$$EQE\ (\%) = \frac{(2n_{HCOO^-} N_A hc)}{(t_{in} \lambda I A)} \times 100 \qquad \text{(S3)}$$

Where n_{HCOO^-} is the number of moles of photogenerated formate, t_{irr} is the irradiation time (s), A is the cross section (m^2), and N_A, h and c are Avogadro's constant (mol^{-1}), the Planck constant (m^2 kg s^{-1}), and the speed of light (m s^{-1}), respectively.

Ion chromatography. IC was carried out on a Metrohm 882 Compact IC Plus ion chromatograph with a conductivity detector. The eluent buffer was an aqueous solution of Na_2CO_3 (3 mM), $NaHCO_3$ (1 mM). Each time, after renewing the eluent buffer the system was calibrated with samples containing 0.1, 0.5, 1, 2, 4 mM of a sodium formate standard diluted in the same buffer as the electrolytic or photocatalytic buffer. Samples were diluted 10× with H_2O and filtered through a 0.2 µm syringe filter before injection in the IC.

X-ray photoelectron spectroscopy. Samples were prepared by drop-casting a suspension of the material in ethanol onto a cleaned FTO slide and dried at 60 ºC: XPS analysis was carried out using an Escalab 250XI spectrometer from Thermo Fisher Scientific (West Sussex, UK). The instrument was operating in constant analyser energy mode. A monochromatic Al-Kα source (1486.74 eV) and a flood gun for charge neutralization were used over an area of approximately 300 μm^2. Survey scans were acquired using a pass energy of 30 eV where 3

scans were recorded using 0.5 eV steps and a dwell of 50 ms. For narrow scans the number of scans was 10 scans for C 1s and O 1s and 70 scans for N 1s, using a pass energy of 30 eV, a step size of 0.05 eV, and a dwell time of 50 ms. Two spectra at room temperature from different areas of the sample were acquired and then results were averaged if more than one point was acquired. The energetic position of the C 1s emission line (binding energy of 284.6 eV) was chosen to calibrate the energy scale of the spectra. CASA XPS software was used to analyse each spectrum, using Gaussian-Lorentzian (30) curve fitting and a Shirley type background.

Scanning electron microscopy. SEM was carried out on a TESCAN MIRA3 FEG-SEM. Cross-sections of the electrodes were prepared and sputtered with a 10 μm layer of Pt prior to the measurement.

Other instrumentation. FT-IR was carried out on a Thermo Scientific Nicolet iS50 FT-IR spectrometer. ^{1}H NMR spectra were recorded on a Bruker DPX-400 MHz spectrometer at room temperature. Chemical shifts are given in ppm and coupling constants in Hz. Chemical shifts for ^{1}H NMR spectra are referenced relative to residual protons in the deuterated solvent (D_2O: ^{1}H = 4.8 ppm, methanol-d_4: ^{13}C = 49.1 ppm. UV–vis spectroscopy was carried out on a Varian Cary 50 UV–vis spectrophotometer using quartz cuvettes with 1 cm path length. The zeta potential was measured using a Malvern Zetasizer Nano ZS. The samples (*a*-CDs or CNTs) were dispersed in H_2O, or the relevant buffer and the pH adjusted using 1 M HCl or 1 M NaOH. All measurements were conducted as three replicates; average results were quoted using the standard deviation as the error. Elemental analysis was carried out by the Microanalysis Service of the Department of Chemistry, University of Cambridge, using an Exeter Analytical CE-440 Elemental Analyzer.

Supplementary figures and tables

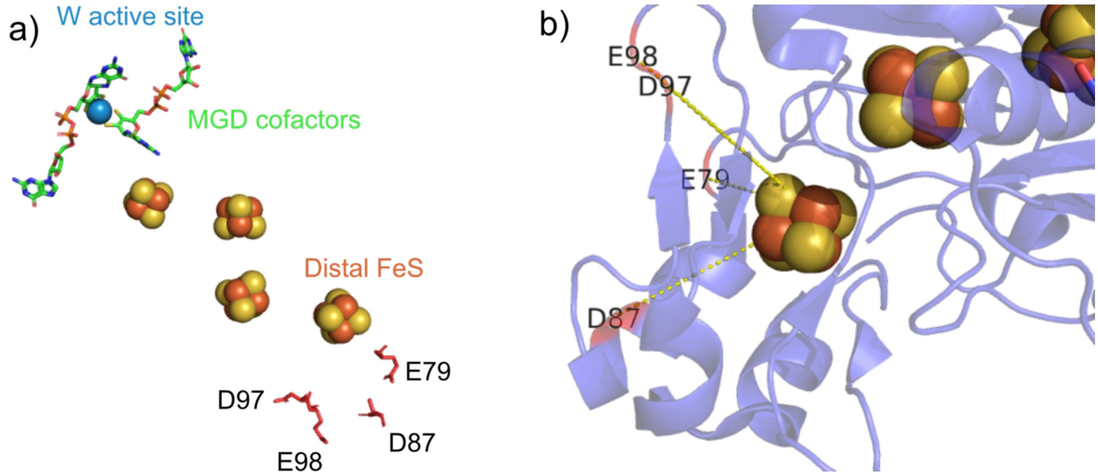

Figure S1. (a) The cofactors of *Dv*H W-FDH (pdb: 6SDV), including the W active site, bis(molybdopterin guanine dinucleotide; MGD) and FeS cofactors, comprising of the distal FeS cluster with the surrounding aspartic acid (D) and glutamic acid (E) amino acid residues. (b) The yellow lines highlight the distance of each negatively charged amino acid from the distal FeS cluster (see Table S2).[1]

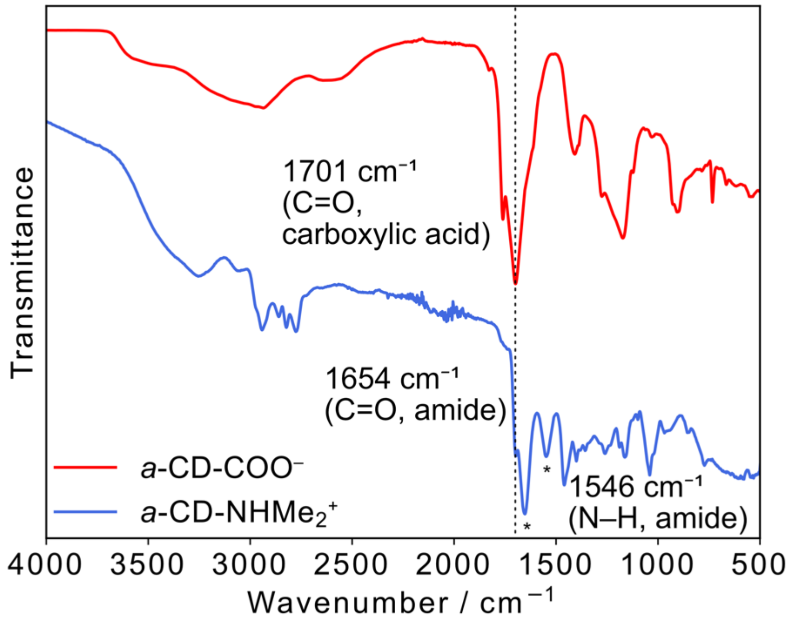

Figure S2. FT-IR spectra of *a*-CD-COO$^-$ and *a*-CD-NHMe$_2^+$. The loss of the carboxylic acid C=O stretch at 1701 cm^{-1} in *a*-CD-COO$^-$ and the appearance of an amide C=O stretch at 1654 cm^{-1} and an N-H bending mode at 1546 cm^{-1} support successful functionalization to *a*-CD-NHMe$_2^+$.

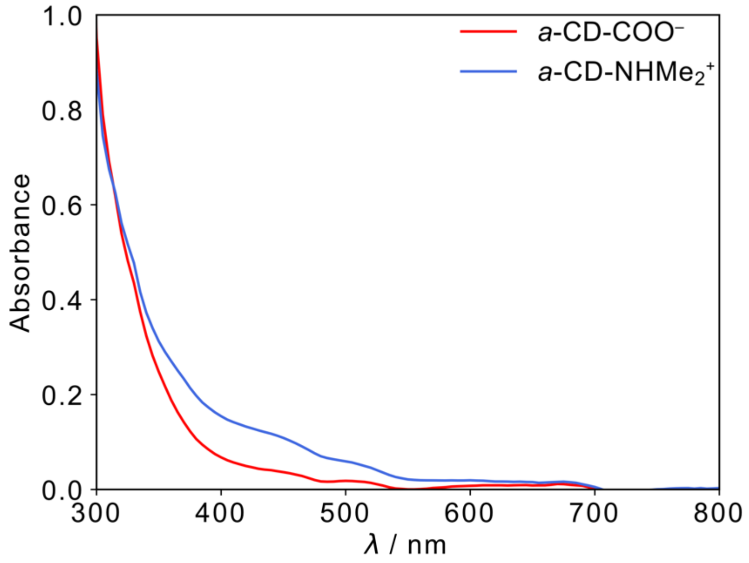

Figure S3. UV-vis absorption spectra for 1 mg mL^{-1} aqueous solutions of *a*-CD-COO^{-} and *a*-CD-$NHMe_2^{+}$ at 25 °C with a cuvette pathlength of 1 cm.

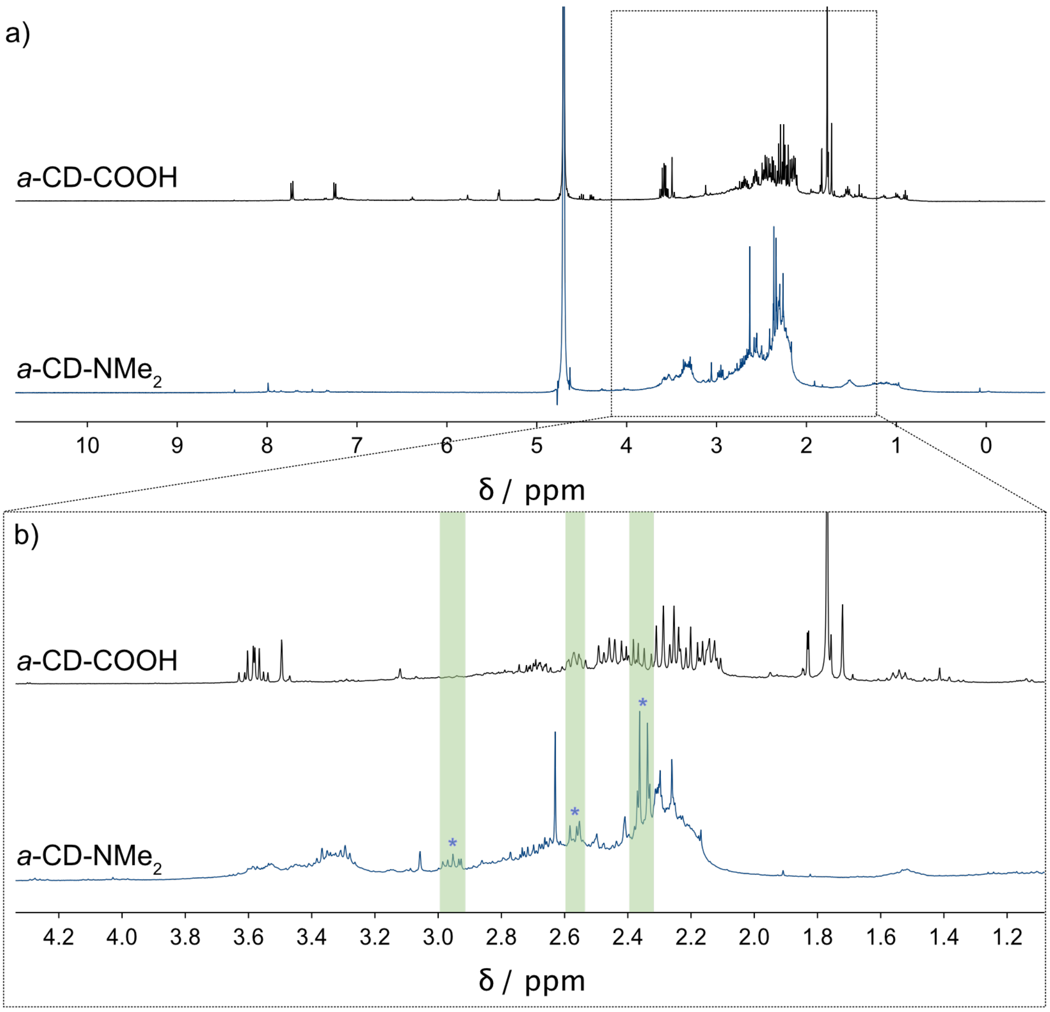

Figure S4. ^{1}H NMR spectra of *a*-CD-COO⁻ and *a*-CD-NHMe$_2^+$ in D_2O. (a) Shows the entire spectra, while (b) shows a zoomed in region where the signals within the shaded region marked by an asterisk in *a*-CD-NHMe$_2^+$ are assigned to the methyl (2.3 – 2.4 ppm) and ethyl backbone (2.5 and 2.9 ppm) proton signals from the -CONH$(CH_2)_2$NH$(CH_3)_2^+$ functional group, which are not present in *a*-CD-COO⁻, consistent with previous reports.[2]

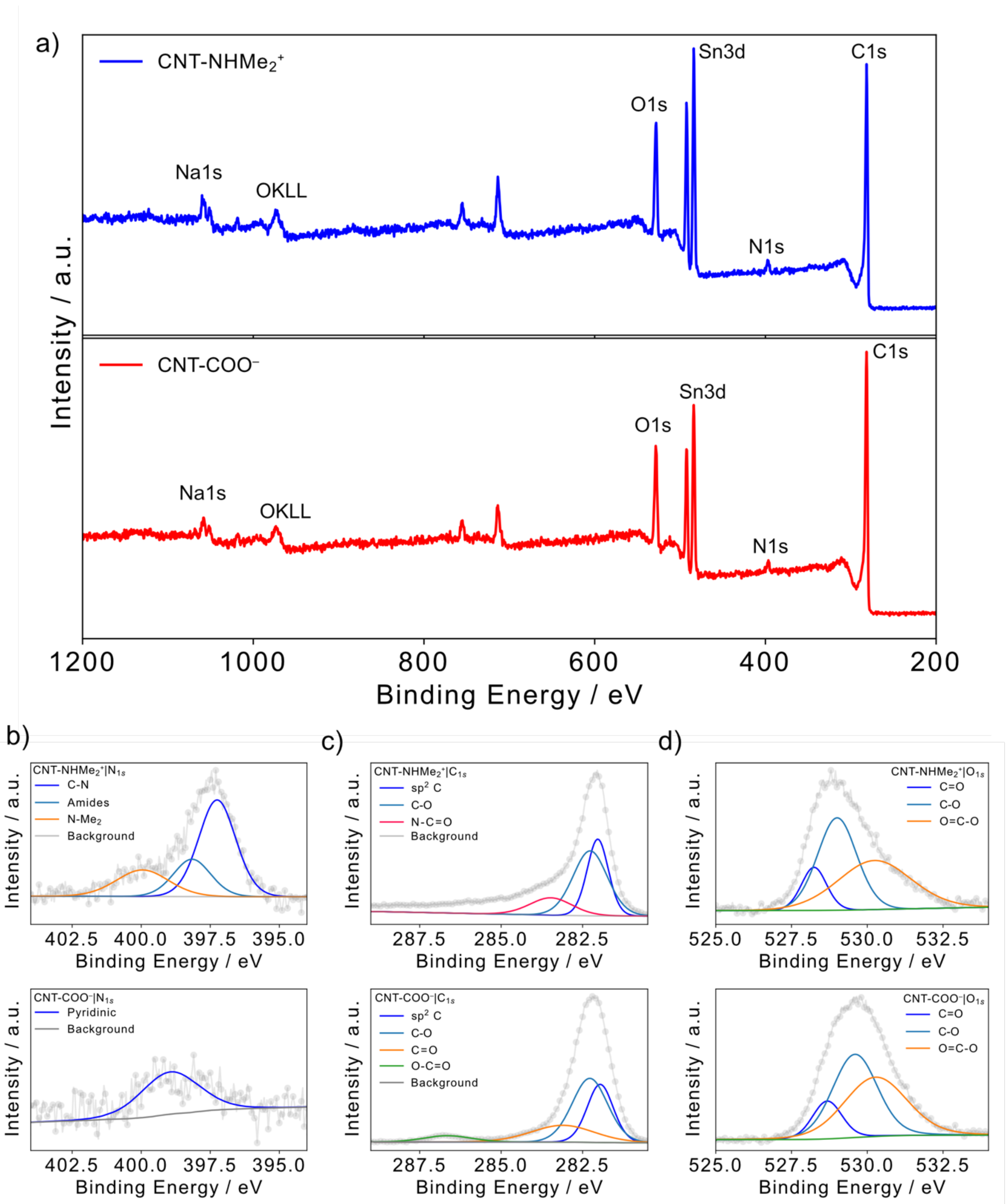

Figure S5. (a) Full XPS survey spectra of CNT-NHMe$_2^+$ (top panel) and CNT-COO$^-$ (bottom panel), (b) deconvolution of the CNT-NMe$_2$ (top panel) and CNT-COO$^-$ (bottom panel) N 1s peaks, showing the presence of a C=O amide (398.2 eV) and a N-Me$_2$ amine (399.5 eV) in CNT-NHMe$_2^+$, (c) deconvolution of the CNT-NHMe$_2^+$ (top panel) and CNT-COO$^-$ (bottom panel) C 1s peaks, (d) deconvolution of the CNT-NHMe$_2^+$ (top panel) and CNT-COO$^-$ (bottom panel) O 1s peaks.

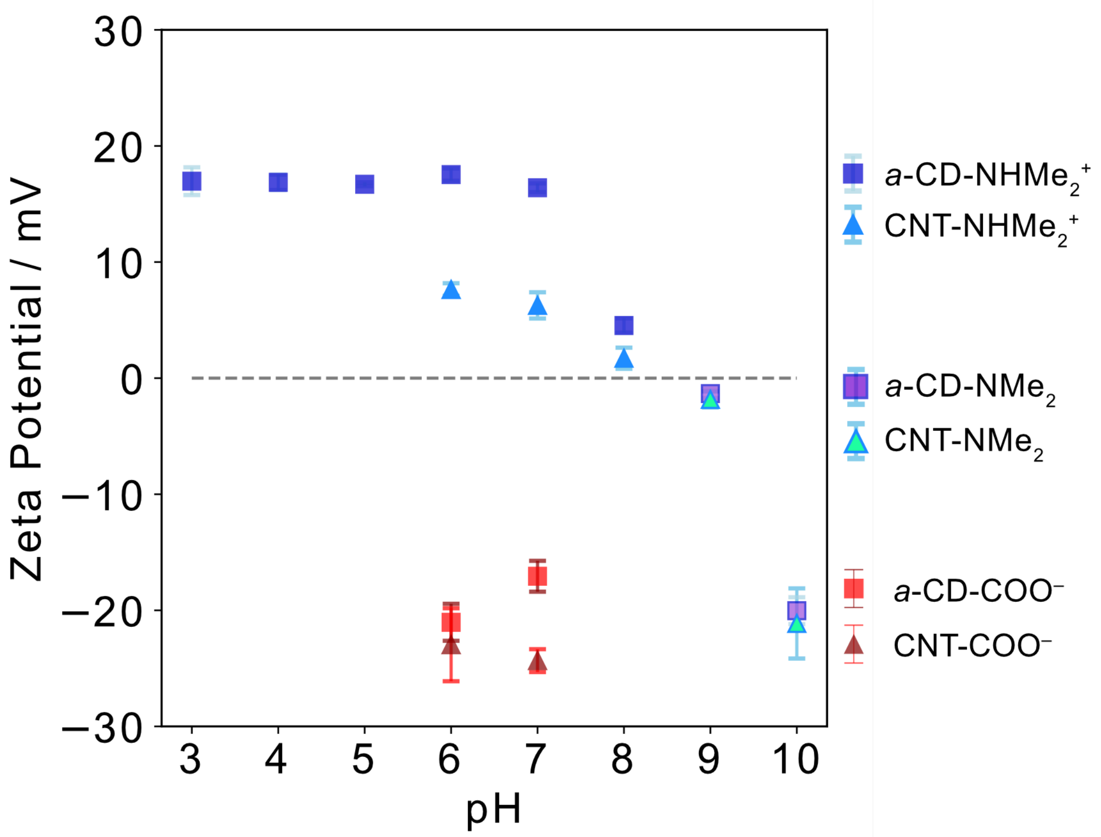

Figure S6. Zeta potential measurements for *a*-CD-$NHMe_2^+$ (1 mg mL^{-1}) and CNT-$NHMe_2^+$ (1 mg mL^{-1}) and *a*-CD-COO^- (1 mg mL^{-1}) and CNT-COO^- (1 mg mL^{-1}) as a function of pH which was adjusted accordingly with 1 M solutions of HCl or NaOH. Error bars represent the standard deviation for a sample size of n = 3.

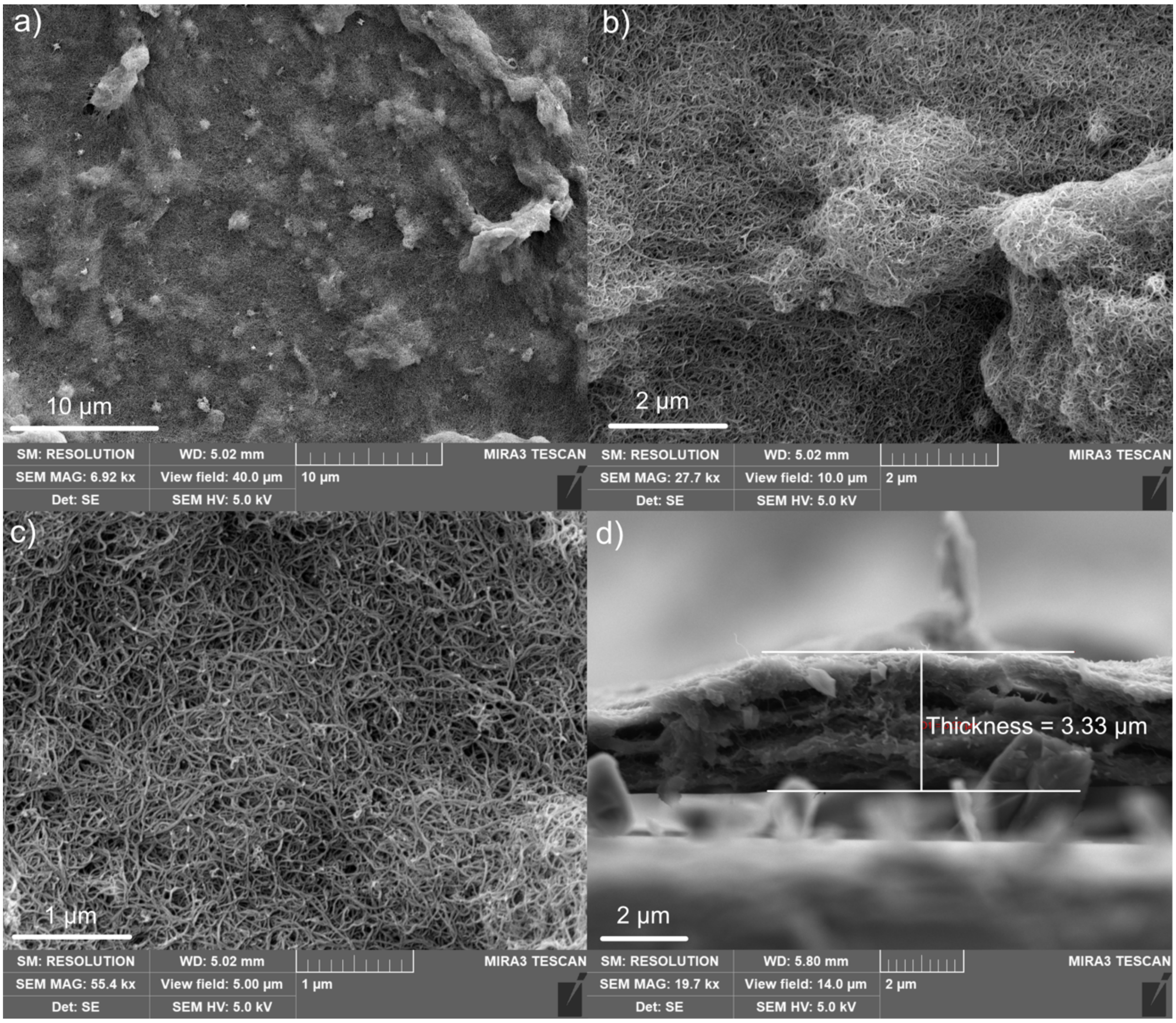

Figure S7. SEM images of a drop-cast CNT film for PFV measurements on a Si substrate (1 mg mL^{-1}) at magnifications of (a) 6.92 thousand times (kx), (b) 27.7 kx, (c) 55.4 kx and (d) a cross-sectional SEM image of the CNT film on a Si substrate at 19.7 kx, highlighting a film thickness of 3.33 μm.

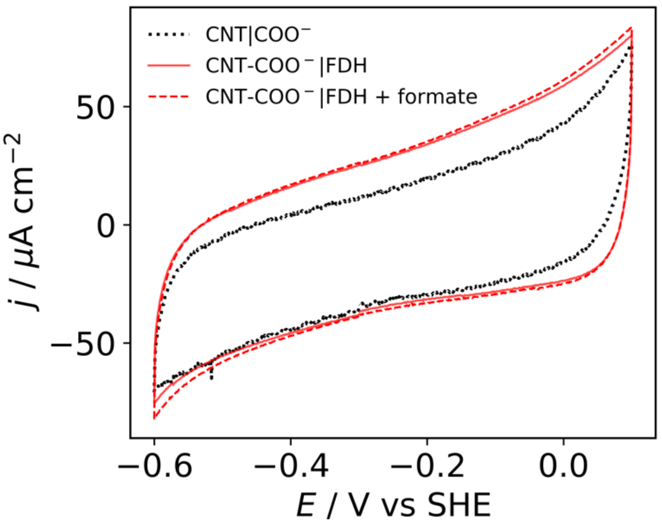

Figure S8. CV scan of an FDH-free CNT-COO⁻ electrode (black dotted line) compared to PFV scans of a FDH immobilized CNT-COO⁻ electrode in 1 atm CO_2 (solid red line) or 1 atm CO_2 and 20 mM sodium formate (dashed red line). Conditions: CNT (15 µg) on a GCE (0.071 cm^2), FDH (40 pmol) immobilized in MOPS (50 mM, pH 7), $NaHCO_3$/KCl electrolyte (100 mM, 50 mM, pH 6.7), 1 atm CO_2, 20 mM sodium formate, v = 5 mV s^{-1}, 25 °C.

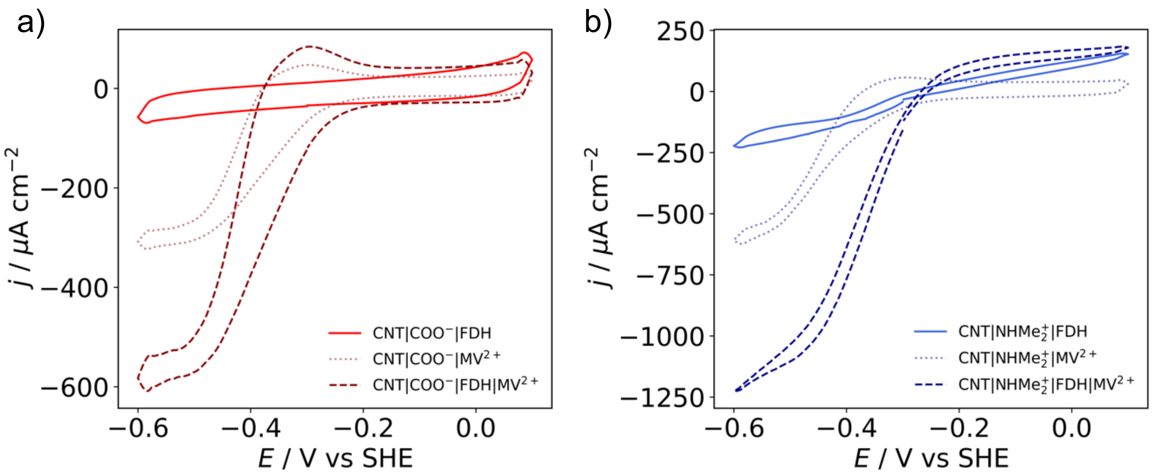

Figure S9. (a) Protein film voltammogram of FDH immobilized on CNT-COO⁻ in the absence (solid red line) or presence (dashed dark red line) of MV^{2+}. (b) Protein film voltammogram of FDH immobilized on CNT-$NHMe_2^+$ in the absence (solid blue line) or presence (dashed dark blue line) of MV^{2+}. The observed increase in catalytic current upon addition of MV^{2+} indicates the presence of adsorbed FDH in an orientation with the distal FeS cluster far from the CNT surface. Dotted traces are background FDH-free CNT electrodes in the presence of MV^{2+}, which shows the reduction of MV^{2+} to $MV^{+\bullet}$. Conditions: CNT (15 µg) on a GCE (0.071 cm^2), FDH (40 pmol) immobilized in MOPS (50 mM, pH 7), $NaHCO_3$/KCl electrolyte (100 mM, 50 mM, pH 6.7), 1 atm CO_2, 20 mM sodium formate, 0.25 mM MV^{2+}, v = 5 mV s^{-1}, 25 °C.

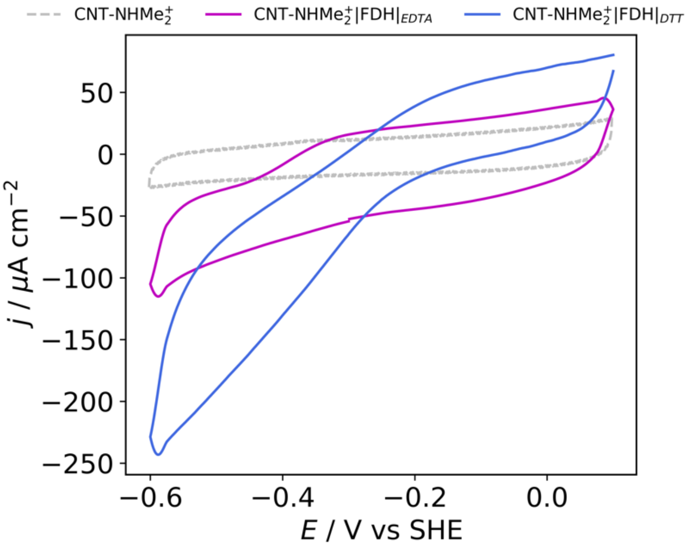

Figure S10. Protein film voltammogram of FDH coimmobilized on CNT-NHMe$_2^+$ in the presence of DTT (blue trace) or EDTA (magenta trace). Conditions: CNT (15 μg) on a GCE (0.071 cm^2), FDH (40 pmol) immobilized in MOPS (50 mM, pH 7) with 10 mM DTT or 10 mM EDTA, in $NaHCO_3$/KCl electrolyte (100 mM, 50 mM, pH 6.7), 1 atm CO_2, $v = 5$ mV s^{-1}, 25 °C. Dashed trace shows the FDH-free control experiment.

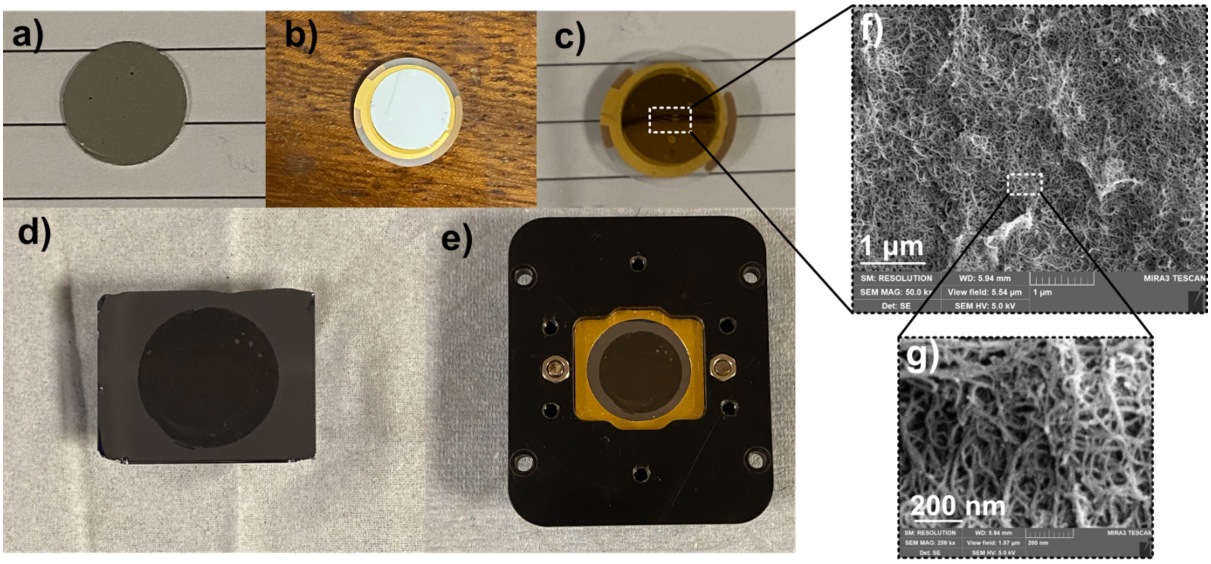

Figure S11. The thin film membrane transfer procedure which begins with (a) filtering an aqueous dispersion of either CNT-COO$^-$ or CNT-NHMe$_2^+$ through a nitrocellulose membrane, (b) placing the membrane face-down on the substrate (QCM chip) and wetting and drying in an oven for 30 mins after which (c) the membrane is dissolved in acetone and methanol, leaving behind the thin CNT membrane on the QCM chip. (d, e) Shows a CNT membrane transferred onto an ATR-IR Si prism by the same method, (f) an SEM image of the CNT film, (g) a zoomed in SEM image of the CNT membrane showing the interwoven mat-like morphology of the CNT membrane.

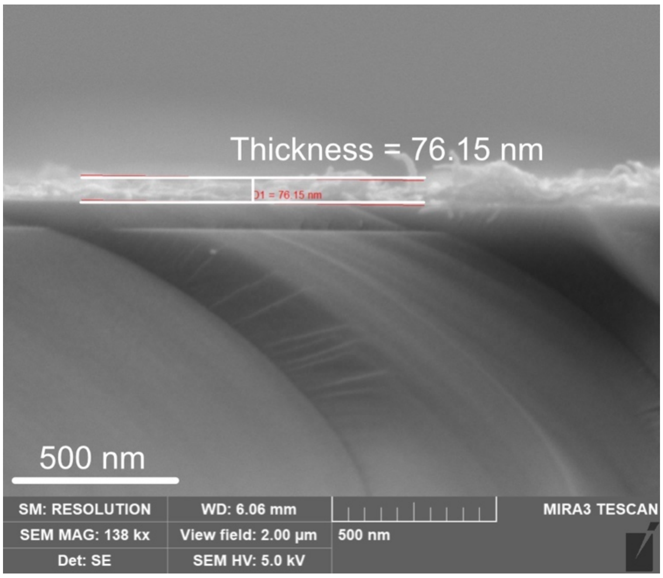

Figure S12. SEM cross-sectional image of the thin CNT membrane transferred to a Si substrate by the membrane transfer method at 138 kx magnification, highlighting a thickness of 76 nm.

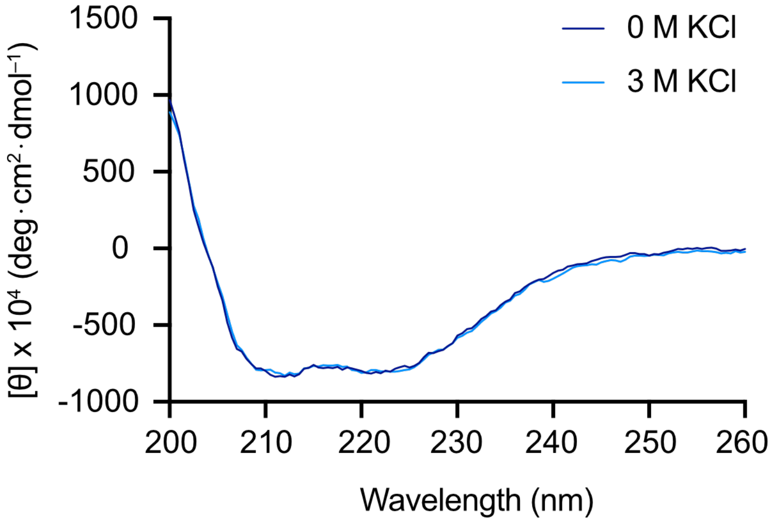

Figure S13. Circular dichroism spectra of the FDH sample before (0 M KCl; dark blue) and after KCl incubation (3 M KCl; light blue) in 20 mM Tris-HCl Buffer, pH 7.6. Spectra were obtained at 20 °C and a 50 nm min^{-1} scan rate.

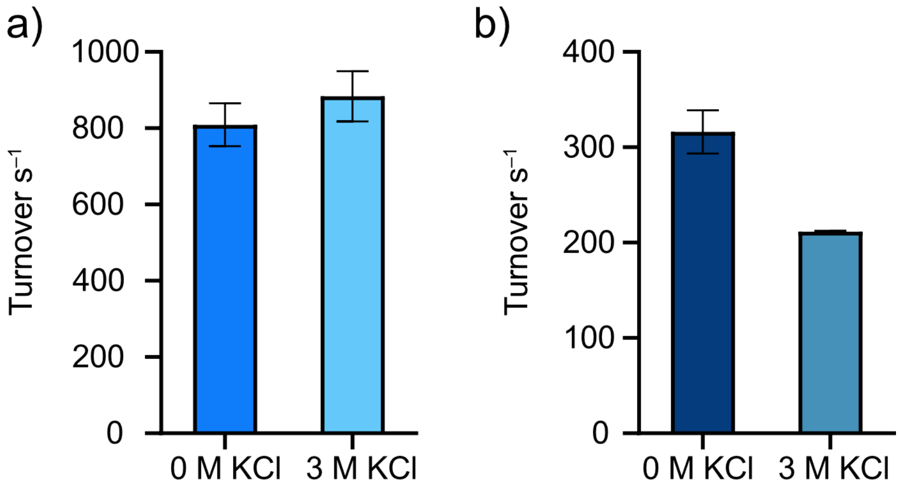

Figure S14. Solution assay activity rates of FDH for (a) formate oxidation and (b) CO_2 reduction before (0 M KCl) and after (3 M KCl) incubation with KCl. FDH was incubated with KCl for 30 mins and removed prior to assaying. Assays were performed in triplicate and error bars represent the standard deviation.

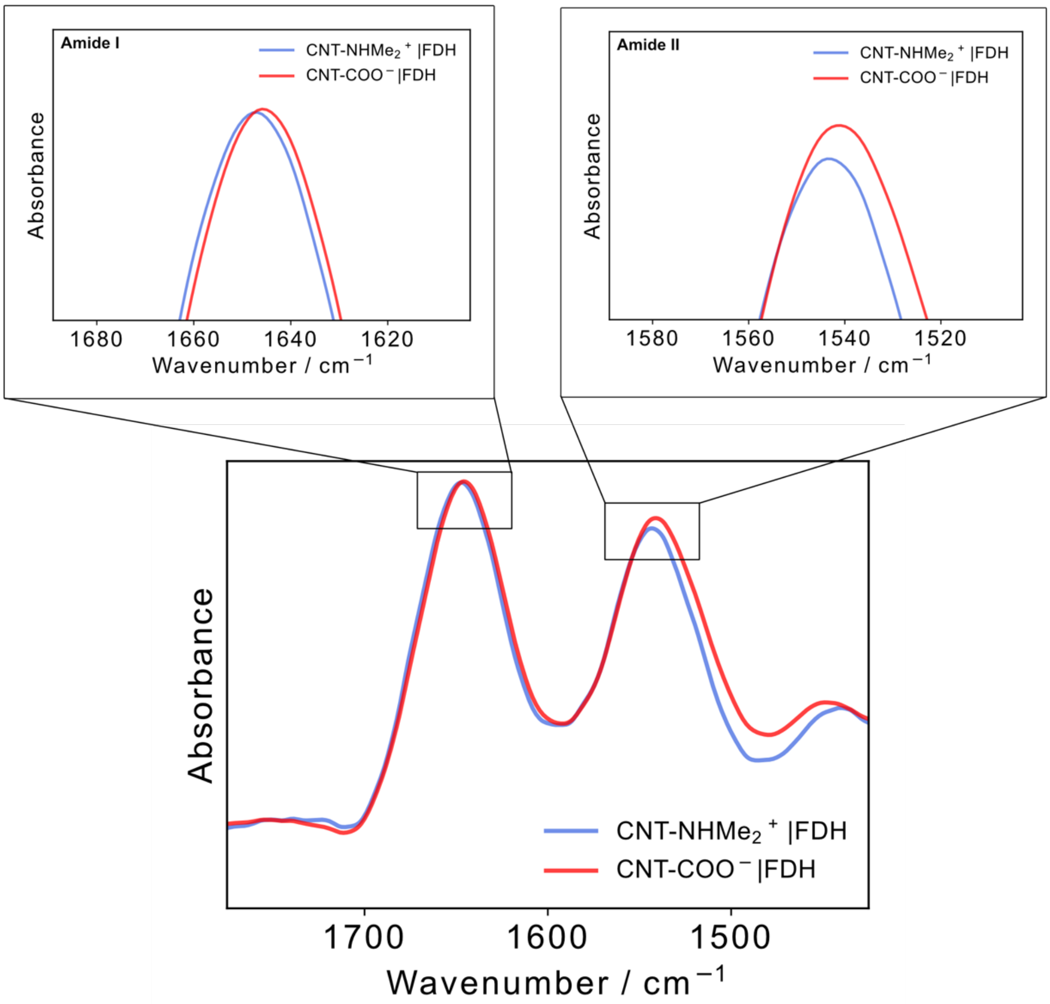

Figure S15. ATR-IR absorbance spectra after 90 mins of the amide band region of FDH adsorption on CNT-$NHMe_2^+$ (blue) and CNT-COO^- (red) coated on a Si prism. Inset is the amide II and amide I bands. Conditions: 400 nM FDH, 50 mM MOPS, pH 7, total volume = 200 µL, 25 °C.

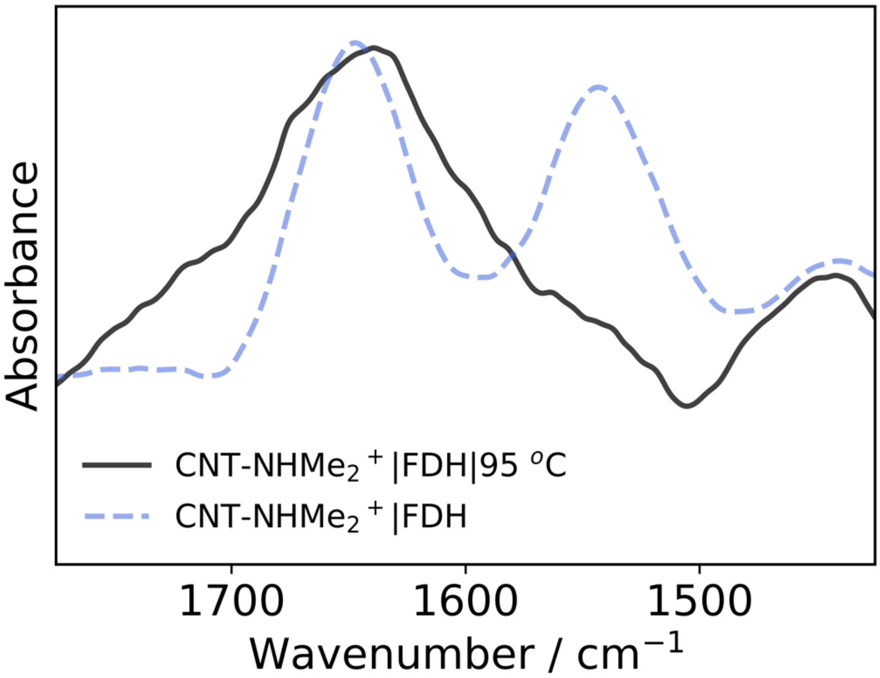

Figure S16. A comparison of the ATR-IR absorbance spectra of FDH adsorbed on a CNT-$NHMe_2^+$ coated Si prism in the active state (blue dashed trace) and in the denatured state (heated to 95 °C, 15 mins, black trace) after 90 mins. The broadening of the amide I and loss of amide II band in the denatured state signals deconformation of the secondary structure of the protein and thus its inactivation. Conditions: 400 nM FDH, 50 mM MOPS, pH 7, total volume = 200 µL, 25 °C.

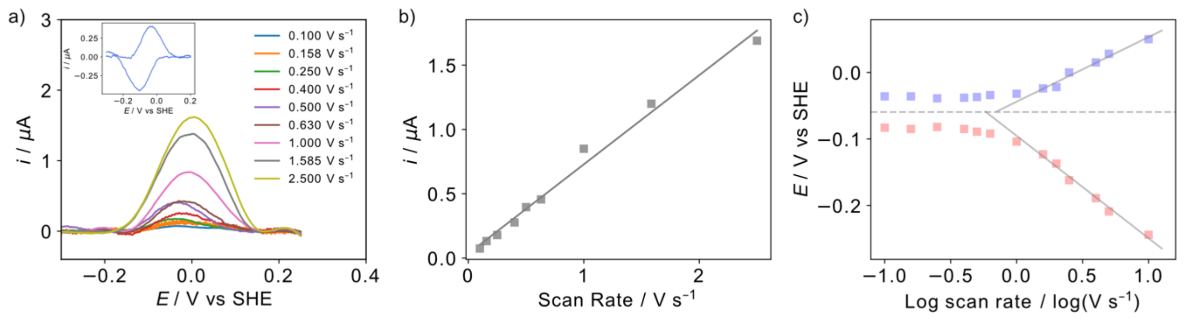

Figure S17. Nonturnover electrochemical signals for determining the electron transfer properties of DET FDH on CNT-NHMe$_2^+$. (a) Background subtracted oxidative peak with E^0 = −0.06 V vs SHE and the background subtracted anodic and cathodic scans at a scan rate of 500 mV s^{-1} (inset). (b) Linear scan rate dependence of the observed signal (gradient = 0.69 ± 0.03 μA V^{-1}, R^2 = 0.99). (c) Scan rate dependence of peak position for determining electron transfer kinetics (α = 0.39 ± 0.01, k_{ET} = 9.7 ± 0.5 s^{-1}). Conditions: CNT-NHMe$_2^+$ (15 μg) on a GCE (0.071 cm^2), FDH (40 pmol) immobilized in MOPS (50 mM, pH 7), MES/KCl (100 mM, 50 mM, pH 6.5), 25 °C.

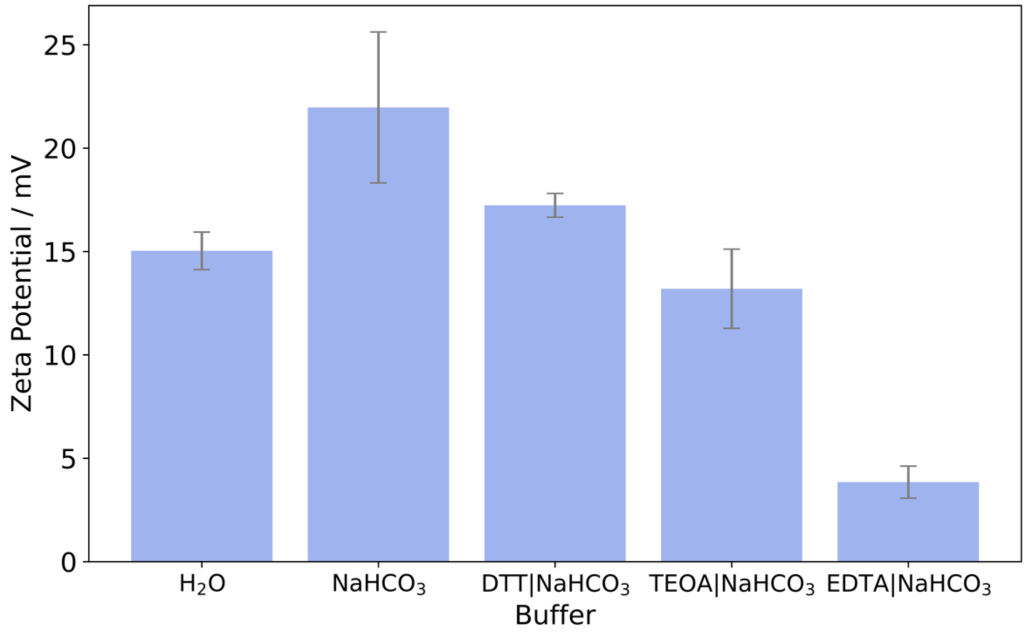

Figure S18. Zeta potential measurements of *a*-CD-NHMe$_2^+$ (1 mg mL^{-1}) in various buffer conditions. Conditions: 100 mM $NaHCO_3$, 10 mM DTT, TEOA or EDTA, 1 atm CO_2, pH 6.7. Error bars represent the standard deviation for a sample size of n = 3.

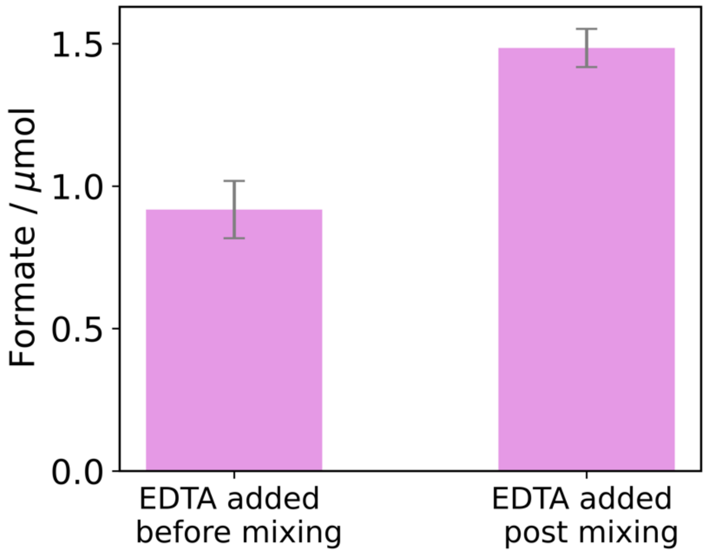

Figure S19. Photocatalytic CO_2 reduction to formate after 24 h with FDH on *a*-CD-$NHMe_2^+$ with EDTA added either before or after the mixing of FDH and *a*-CD-$NHMe_2^+$ in $NaHCO_3/CO_2$. Conditions: 40 pmol FDH, 10 mM electron donor EDTA, 1 mg mL^{-1} *a*-CD-$NHMe_2^+$, 100 mM $NaHCO_3$, 1 atm CO_2, pH 6.7, 25 °C, total volume = 1 mL, assembled in an anaerobic glovebox, simulated solar-light irradiation: AM 1.5G, 100 mW cm^{-2}. Error bars represent the standard deviation for a sample size of n = 3.

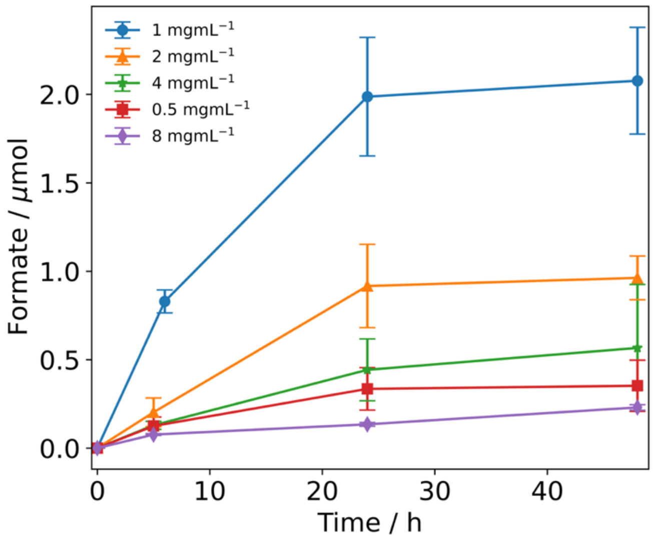

Figure S20. Photocatalytic CO_2 reduction to formate with FDH immobilized on *a*-CD-$NHMe_2^+$ under different concentrations of *a*-CD-$NHMe_2^+$. Conditions: 40 pmol FDH, 10 mM DTT, 100 mM $NaHCO_3$, 1 atm CO_2, pH 6.7, 25 °C, total volume = 1 mL, assembled in an anaerobic glovebox, simulated solar-light irradiation: AM 1.5G, 100 mW cm^{-2}. Error bars represent the standard deviation for a sample size of n = 3.

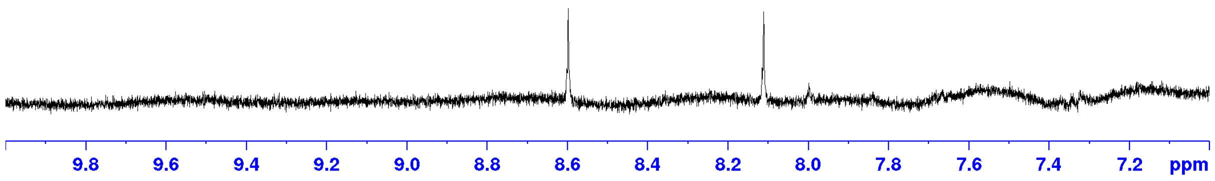

Figure S21. ^{1}H-NMR spectrum (400 MHz, H_2O:D_2O, 90:10) of the ^{13}C-formate product derived from the photocatalytic CO_2 reduction with $NaH^{13}CO_3$ (N_2, pH 6.7) showing the splitting of the ^{13}C formate signals after 24 h photocatalysis due to the coupling of ^{1}H with ^{13}C.

Table S1. Quantitative comparison of the elemental composition of the different CNT and *a*-CD samples.

Sample	%C	%H	%N
CNT-COO^{-}	86.92	0.49	0.23
CNT-$NHMe_2^{+}$	71.06	4.38	**1.77**
a-CD-COO^{-}	52.64	5.31	0.00
a-CD-$NHMe_2^{+}$	53.68	8.73	**14.94**

Table S2. A comparison of the distances of selected negatively charged aspartic acid (E) and glutamic acid (D) amino acid residues from the distal FeS cluster.

Residue	Distance from distal FeS (Å)
E79	7.8
D87	12.5
D97	11.4
E98	13.6

Table S3. Comparison of TOFs for DET and MET photocatalytic CO_2 reduction systems with FDH.

System	TOF / h^{-1}	Reference
***a*-CD-$NHMe_2^{+}$|FDH**	**3.5×10^3 (6h)**	**This work**
RuP|TiO_2|FDH	3.9×10^4 (6h)	7
DPP|TiO_2|FDH	1.7×10^4 (6h)	7
FDH|MOF|Rh mediator|perylene dye	865 (24h)	8
Graphene-based photocatalyst	1.69 (2h)	9
Zinc porphyrin photocatalyst	0.10 (3h)	10
Photosensitization of Mg chlorophyll-a	4.64 (1h)	11
Viologen-based artificial co-enzyme	9.38 (0.17 h)	12

Supplementary references

1. Oliveira, A. R.; Mota, C.; Mourato, C.; Domingos, R. M.; Santos, M. F. A.; Gesto, D.; Guigliarelli, B.; Santos-Silva, T.; Romão, M. J.; Cardoso Pereira, I. A., Toward the Mechanistic Understanding of Enzymatic CO_2 Reduction. *ACS Catal.* **2020,** *10* (6), 3844-3856.
2. Hutton, G. A.; Reuillard, B.; Martindale, B. C.; Caputo, C. A.; Lockwood, C. W.; Butt, J. N.; Reisner, E., Carbon Dots as Versatile Photosensitizers for Solar-Driven Catalysis with Redox Enzymes. *J. Am. Chem. Soc.* **2016,** *138* (51), 16722-16730.
3. Guo, C. X.; Zhao, D.; Zhao, Q.; Wang, P.; Lu, X., Na(+)-functionalized carbon quantum dots: a new draw solute in forward osmosis for seawater desalination. *Chem. Commun.* **2014,** *50* (55), 7318-21.
4. Martindale, B. C.; Hutton, G. A.; Caputo, C. A.; Reisner, E., Solar hydrogen production using carbon quantum dots and a molecular nickel catalyst. *J. Am. Chem. Soc.* **2015,** *137* (18), 6018-25.
5. Wu, Z.; Chen, Z.; Du, X.; Logan, J. M.; Sippel, J.; Nikolou, M.; Kamaras, K.; Reynolds, J. R.; Tanner, D. B.; Hebard, A. F.; Rinzler, A. G., Transparent, conductive carbon nanotube films. *Science* **2004,** *305* (5688), 1273-6.
6. Sauerbrey, G., Verwendung von Schwingquarzen zur Wägung dünner Schichten und zur Mikrowägung. *Zeitschrift für Physik* **1959,** *155* (2), 206-222.
7. Miller, M.; Robinson, W. E.; Oliveira, A. R.; Heidary, N.; Kornienko, N.; Warnan, J.; Pereira, I. A. C.; Reisner, E., Interfacing Formate Dehydrogenase with Metal Oxides for the Reversible Electrocatalysis and Solar-Driven Reduction of Carbon Dioxide. *Angew. Chem. Int. Ed.* **2019,** *58* (14), 4601-4605.
8. Chen, Y.; Li, P.; Zhou, J.; Buru, C. T.; Dordevic, L.; Li, P.; Zhang, X.; Cetin, M. M.; Stoddart, J. F.; Stupp, S. I.; Wasielewski, M. R.; Farha, O. K., Integration of Enzymes and Photosensitizers in a Hierarchical Mesoporous Metal-Organic Framework for Light-Driven CO_2 Reduction. *J. Am. Chem. Soc.* **2020,** *142* (4), 1768-1773.
9. Yadav, R. K.; Baeg, J. O.; Oh, G. H.; Park, N. J.; Kong, K. J.; Kim, J.; Hwang, D. W.; Biswas, S. K., A photocatalyst-enzyme coupled artificial photosynthesis system for solar energy in production of formic acid from CO_2. *J. Am. Chem. Soc.* **2012,** *134* (28), 11455-61.
10. Miyatani, R.; Amao, Y., Photochemical synthesis of formic acid from CO_2 with formate dehydrogenase and water-soluble zinc porphyrin. *J. Mol. Catal., B Enzym* **2004,** *27* (2-3), 121-125.
11. Tsujisho, I.; Toyoda, M.; Amao, Y., Photochemical and enzymatic synthesis of formic acid from CO_2 with chlorophyll and dehydrogenase system. *Catal. Commun.* **2006,** *7* (3), 173-176.
12. Ikeyama, S.; Amao, Y., An Artificial Co‐enzyme Based on the Viologen Skeleton for Highly Efficient CO_2 Reduction to Formic Acid with Formate Dehydrogenase. *ChemCatChem* **2017,** *9* (5), 833-838.

End of Supporting Information
